# Transcript Analysis and Regulative Events during Flower Development in Olive (*Olea europaea* L.)

**DOI:** 10.1371/journal.pone.0152943

**Published:** 2016-04-14

**Authors:** Fiammetta Alagna, Marco Cirilli, Giulio Galla, Fabrizio Carbone, Loretta Daddiego, Paolo Facella, Loredana Lopez, Chiara Colao, Roberto Mariotti, Nicolò Cultrera, Martina Rossi, Gianni Barcaccia, Luciana Baldoni, Rosario Muleo, Gaetano Perrotta

**Affiliations:** 1 Institute of Biosciences and Bioresources, National Research Council, Perugia, Italy; 2 Laboratory of Molecular Ecophysiology and Biotechnology of Woody Plants, Department of Agricultural and Forestry Science, University of Tuscia, Viterbo, Italy; 3 Laboratory of Plant Genetics and Genomics, DAFNAE, University of Padova, Legnaro (PD), Italy; 4 Italian National Agency for New Technologies, Energy and Sustainable Economic Development, TRISAIA Research Center, Rotondella (MT), Italy; University of Perugia, ITALY

## Abstract

The identification and characterization of transcripts involved in flower organ development, plant reproduction and metabolism represent key steps in plant phenotypic and physiological pathways, and may generate high-quality transcript variants useful for the development of functional markers. This study was aimed at obtaining an extensive characterization of the olive flower transcripts, by providing sound information on the candidate MADS-box genes related to the ABC model of flower development and on the putative genetic and molecular determinants of ovary abortion and pollen-pistil interaction. The overall sequence data, obtained by pyrosequencing of four cDNA libraries from flowers at different developmental stages of three olive varieties with distinct reproductive features (Leccino, Frantoio and Dolce Agogia), included approximately 465,000 ESTs, which gave rise to more than 14,600 contigs and approximately 92,000 singletons. As many as 56,700 unigenes were successfully annotated and provided gene ontology insights into the structural organization and putative molecular function of sequenced transcripts and deduced proteins in the context of their corresponding biological processes. Differentially expressed genes with potential regulatory roles in biosynthetic pathways and metabolic networks during flower development were identified. The gene expression studies allowed us to select the candidate genes that play well-known molecular functions in a number of biosynthetic pathways and specific biological processes that affect olive reproduction. A sound understanding of gene functions and regulatory networks that characterize the olive flower is provided.

## Introduction

Olive (*Olea europaea* L.) is a wind pollinated, andromonoecious species whose cluster inflorescences are paniculate (approaching compound botryoids [1], and whose flower position on the inflorescence may affect its development and fertility [2]. The hermaphrodite flower consists of a small, greenish calyx, four white petals, two stamens with large anthers and one single pistil with a bilobed stigma, a short style and a bilocular ovary with four ovules [3]. In the Mediterranean area, floral initiation occurs at beginning of winter on one-year-old shoots, and floral buds and flower primordia are not evident until March, two months before anthesis [3]. Blooming occurs in late spring, typically at the end of May, and lasts for approximately two weeks.

Complex molecular and cellular processes occur throughout flower development and are required for the proper regulation of cell proliferation and expansion, the development of reproductive tissues and the sculpting of the final form of the different organs [4]. Studies on the genetic determinants of flower development and plant reproduction have not yet been conducted in olive; however, the results from other plants provided a wide variety of candidates that might also have utility in this species. The analysis of homeotic mutants in *Arabidopsis thaliana* and *Antirrhinum majus* resulted in the formulation of the genetic ABC model, which, in its advanced conformation, predicts the combinatorial action of five classes of genes (ABCDE), most of which encode MADS-box transcription factors, controlling the identity of the floral organ types: sepals, petals, stamens, carpels and ovules [5, 6]. In the last two decades, studies on MADS-box genes disclosed the importance of these determinants in flower and fruit development of different plant species, as they exert a wide range of functions, including the development of reproductive structures, the control of flowering time and vegetative development [7]. Some varieties are characterized by a high number of male flowers [8, 2], due to a high rate of ovary abortion and pistil desiccation [9, 10, 11]. The incidence of pistil abortion is influenced by nutritional or stress conditions [12, 13], and it has been proposed that starch and sucrose metabolism might have a role in this process [10]. Starch accumulates in the plastids of all of the components of flower whorls until the beginning of anthesis [14]. In perfect flowers, starch grains are observable in the stigma, style, ovary wall and ovule integument, while starch is not detected in the pistil of staminate flowers, suggesting a correlation with the ovary abortion of olive flowers [10].

The occurrence of male flowers in olive shares several analogies with the high incidence of androdioecious flowers in *Phillyrea angustifolia*, another member of the Oleaceae family [15]. In addition to flowers with aborted ovaries, male-sterility may also occur in olive [16], which is likely controlled at cytoplasmic level [17]. However, self-incompatibility and inter-incompatibility represent the most important reproductive barriers in olive, whose mechanisms act to reduce the fruit set and crossing chances [18, 19, 20]. The system of incompatibility of olive is still unknown, but the first evidence of the occurrence of a sporophytic system has been recently provided [21, 22, 23]. In self-incompatible plants, the main recognition step is accomplished by the interaction between female and male determinants, which are usually encoded at a single polymorphic locus (S-locus) [24]. Subsequently, appropriate cell signalling and cell-cell communication are crucial for pollen acceptance and growth, requiring complex intercellular communication between the male and female cells. Pollen tube growth occurs directionally through the stigma and style to enter the ovary, and is influenced by chemotropic agents, as well as a variety of lipids, ions, proteins and metabolites that are produced by the pistil [25]. Various proteins that are involved in different steps and mechanisms of pollen-pistil interactions have been identified and characterized in plants [26, 27].

All of these regulative pathways are of crucial interest in olive reproduction because a single tree may produce as many as 500,000 flowers, but only a small percentage of them (1–2%) may set fruits due to various factors, such as wind pollination syndrome, flower development defects (*i*.*e*., ovary abortion), plant reproductive barriers (i.*e*., self-incompatibility and male-sterility) and competition for maternal nutritional resources [28, 11, 29].

Massive EST sequence collections from large-scale transcriptomic analyses of different olive organs, such as the fruits, leaves, stems, buds and roots [30, 31, 32, 33, 34], and the representative transcriptomes of pollen and pistils that have recently been released [35], have provided the first pictures of olive gene functions and opened the door to understanding the regulatory networks that characterize the important metabolic pathways. Gene expression profiling through microarray-based analysis allowed us to highlight the roles played by nutrition metabolism, hormonal control and the flowering process on alternate bearing in olive [36].

This study extensively characterizes the olive flower transcriptomes by focusing on the genetic bases that control flower development, sex differentiation, the mating system, pollination and the loss of female function, which represent processes that have largely been unexplored in this species, despite their impact on the olive agronomic performance. Hence, this manuscript reports the first characterization of the genes expressed during anthesis in genotypes characterized by a different rate of staminate flowers and fruit set after self-pollination, providing a significant advance in the understanding of the molecular mechanisms regulating flower development and fruit set in *Olea europaea*.

## Materials and Methods

### Plant material

**Fig 1:**
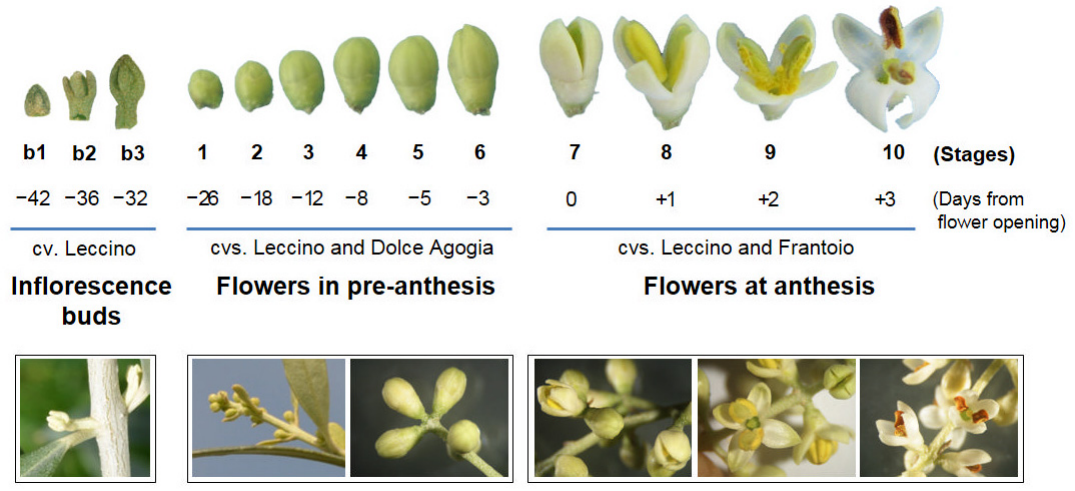
Stages of olive flower development corresponding to the sampling dates. The sampling period includes the differentiation of flower clusters from inflorescence buds and the subsequent stages until the anther dehiscence of the developed flowers. Pictures of entire flower clusters (below) and single flower buds (above) are shown. Stages 1–6: developing flowers at pre-anthesis (26 to 3 days before anthesis). Stages 7–10: flowers at anthesis (0 to 3 days after anthesis). Stages b1-b3 represent the inflorescence buds collected at 42, 36 and 32 days before anthesis and were only used for the RT-qPCR experiments on the candidate homeotic genes.

Olive flowers (Fig 1) were sampled from the plants of self-incompatible (Leccino and Dolce Agogia) and self-compatible (Frantoio) cultivars. Dolce Agogia also represents a highly staminate flower cultivar. The Leccino and Frantoio cultivars exhibit null and very high rates of fruit set under self-pollination conditions, respectively. In fact, Leccino is known to be completely self-incompatible [37], whereas Frantoio is considered partially self-compatible [22]. The flowers for the sequencing experiments were collected from adult trees established at the Olive Cultivar Collection of CREA—The Olive Growing and Olive Product Industry Research Centre (Spoleto, Perugia, Italy).

To ensure receptive stigmas free of contaminant pollen, twigs with inflorescences were bagged three weeks before stigmas became receptive using duraweb pollination bags 63 x 45 cm (PBS International) sealed with adhesive paper. This allowed self-pollen to reach the stigma in both the self-compatible (Frantoio) and self-incompatible varieties (Leccino and Dolce Agogia). The samples were collected at different stages, from the very early developmental stages when the ovaries and stamens are developing (26, 18, 12, 8, 5 and 3 days before anthesis, respectively, which correspond to stages 1, 2, 3, 4, 5 and 6 reported in Fig 1) for Leccino and Dolce Agogia, to anthesis and anther dehiscence, when the pollen grains interact with the pistils (0, 1, 2, 3 days after anthesis, which correspond to stages 7, 8, 9 and 10, respectively) for Leccino and Frantoio. Three plants per genotype were considered and about 40 flowers were collected from each plant, per each flower developmental stage. The samples were pooled into four distinct sets: L1-6: Leccino pre-anthesis (stages 1–6); L7-10: Leccino anthesis (stages 7–10); DA1-6: Dolce Agogia pre-anthesis (stages 1–6); and F7-10: Frantoio anthesis (stages 7–10). Only the leading flowers of the apical and sub-apical clusters were included in the pool, based on the higher probability of obtaining perfect flowers [3]. According to Reale et al. [10], hermaphrodite flowers at stage 1, which is approximately five to six weeks before anthesis, possess anthers where only the exothecium is differentiated and the carpels are at the initial stage in the gynoecium. At approximately four weeks before flower opening, the microspore mother cells start their development in the anthers; however, the carpels are not yet differentiated and the nucellus and integument become evident in the ovules. At stages 2–6, the microspore and megaspore mother cells are produced, a callose wall begins to be formed, pistil differentiates into style and stigma, whereas, in staminate flowers, pistil differentiation arrests. At the beginning of anthesis (stage 7), the bisporic embryo sac is completely differentiated and the microgametogenesis forms pollen grains. At full anthesis (stages 8–10), the anthers initiate dehiscence, the pollen grains are released to the stigmas, and the processes characterizing pollen tube emission and growth occur.

For the RT-qPCR analyses, the entire flowers or single flower parts (calix and corolla, anthers, and pistil) of Leccino, Dolce Agogia and Frantoio were collected as described above. Different samples were used to analyse the different sets of genes according to their putative function. In particular, the RT-qPCR analyses on the genes encoding key proteins in flower development were performed on the calyx-corolla, anthers and pistils of flowers at stage 4, the whole inflorescence buds collected before flower cluster differentiation, 42, 36 and 32 days before anthesis (stages b1, b2 and b3, respectively, Fig 1), and the differentiated flowers of Leccino at progressive developmental stages (stages 1, 2 and 3). The pistils and anthers from perfect flowers, and anthers from staminate flowers of Leccino and Dolce Agogia at stages 6 and 9 were used to analyse the genes that are putatively involved in ovary abortion. For this analysis, the entire undeveloped flowers (stage 9), which are usually positioned at the base of the flower clusters, were also investigated because they may represent flowers that are unable to open, likely due to incomplete differentiation of the floral organs. RT-qPCR analyses of the genes involved in pollen-pistil interactions were performed using the anthers and pistils at anthesis (stages 7, 8, 9 and 10) and the anthers collected before anthesis (stage 5) and at anthesis (stages 7 and 8) from Leccino and Frantoio.

### cDNA synthesis and 454 sequencing

The total RNA was extracted from each flower sample using an RNeasy Plant Mini Kit (Qiagen, Hilden, Germany). The contaminating genomic DNA was removed by DNase I (Qiagen, Hilden, Germany) treatment. The cDNAs were prepared using the SMART PCR cDNA Synthesis kit (Clontech, Palo Alto, CA, USA). First strand synthesis was performed using 8 μg of the total RNA, as described by [30]. The double stranded cDNAs were purified using the QIAquick PCR purification kit (Qiagen, Hilden, Germany) and quantified with a fluorimeter (Victor 2, Perkin Elmer, Wellesley, MA, USA). To verify the cDNA quality and fragment length, all samples were separated on a 1.5% agarose gel.

Approximately 5 μg of each cDNA sample were sheared into small fragments via nebulization. The four shotgun cDNA libraries were sequenced using a 454 GS FLX+ Titanium Sequencer (Roche Diagnostics Corporation, Basel, Switzerland). The raw reads were archived in the NCBI SRA database (Accession number: SRP066775).

### Bioinformatic EST processing protocol

The raw 454 reads were trimmed to exclude the adaptors and primers used to prepare the library, and then assembled using the GS De Novo Assembler Software (Roche Diagnostics Corporation, Basel, Switzerland).

Functional annotation of the unigenes (contigs and singletons) was based on comparisons with the NCBI non-redundant (nr) database using BLASTx (significance threshold: E-value ≤ 1-e^-5^). Moreover, the GI identifiers (http://www.ncbi.nlm.nih.gov/) of the best BLASTx hits were mapped to the UniProtKB protein database (http://www.uniprot.org/) to extract the Gene Ontology (GO, http://www.geneontology.org/) terms for further functional annotations. To identify the main biological pathways active in olive flowers, we mapped the detected genes to the reference canonical pathways in the KEGG database using the KO identifier (http://www.genome.jp/kegg/), which is a classification of the orthologous genes defined by KEGG. Computational annotation of the differentially expressed EST datasets was performed using the Blast2GO software v1.3.3 (http://www.blast2go.org) [38], as described by Galla et al. [31].

To assess the relative abundance of the gene transcripts between the cDNA samples, the statistical R test was applied [39]. This test allowed to normalize the data based on the total number of reads per each library. All contigs with R>8 (true positive rate of ~98%) were considered as differentially expressed. Multivariate analyses, such as the Hierarchical Clustering Analysis (HCA) and Principal Component Analysis (PCA), were performed using GeneSpring version 7.3 (Agilent, Santa Clara, CA, USA).

Functional class scoring was implemented using a MapMan (http://mapman.gabipd.org/web/guest/mapman) analysis on the olive datasets, which were properly rearranged as input files. Briefly, the *Arabidopsis* proteome was downloaded from TAIR (ftp://ftp.arabidopsis.org/home/tair/Sequences/blast_datasets/TAIR10_blastsets/) and used as the reference database for local BLASTX analysis, with the *O*. *europaea* transcriptome dataset as the query. The AGI codes relative to the putative homologous *Arabidopsis* genes (E-value cut-off: 1-e^-6^) were recovered by each tabular BLAST result (BLAST-2.2.25+ argument: -outfmt 7) and used to download the correct ATH1-121501 GeneChip identifiers (Affymetrix) from TAIR (http://www.arabidopsis.org/tools/bulk/microarray/index.jsp). The data were expressed as reads per kilobase of gene length per million reads (rpkm).

### Analysis of the transcripts encoding key proteins in flower development according to the ABC model

A self-confirming double bioinformatic approach was preliminarily adopted to identify the candidate ABCDE genes in olive using sequences from *Arabidopsis thaliana*, *Antirrhinum majus*, *Petunia hybrida*, *Solanum lycopersicum*, *Populus trichocarpa*, *Vitis vinifera*, *Malus domestica* and *Prunus persica* as references [40]. Briefly, separate ClustalW alignments were performed for each class of MADS-box genes with CLC Sequence viewer 6 (http://www.clcbio.com) and were used to construct the HMM patterns for searching the target databases with HMMER v3.0 (available at http://www.hmmer.org). The olive proteome deduced from our EST dataset was then used as a target dataset for both HMM- and BLASTp-based searches using the Arabidopsis sequences as queries. The results from both methods were finally cross-checked to discard possible false-positives.

The phylogenetic tree was constructed with the Neighbour-Joining method using the MEGA software (version 5.2) [41]. The p-distance model and the pairwise deletion option were adopted to construct the Neighbour-Joining tree. A bootstrap analysis was performed by applying 1,000 replications. The list of all proteins included in the phylogenetic analysis and their relative accession numbers is reported in S1 Table.

### Analysis of the putative orthologous genes involved in ovary abortion and pollen-pistil interactions

The amino acid sequences of the functionally characterized proteins from other plant species that are involved in ovary abortion (starch and sucrose metabolism, polyamine biosynthesis, flower carpel differentiation, programmed cell death, and regulation of polyphenol biosynthesis) and pollen-pistil interactions (lipid transfer proteins, cysteine-rich extracellular proteins, NTP family proteins, pectin methylesterases, oleosins, β-1,3-glucanases, incompatibility determinants, etc.) were used as query to search putative olive orthologues in the local olive libraries using a tBLASTn-based approach and the BioLign software v4.0 (http://biolign.software.informer.com/4.0/). The sequences that met the cut-off value of 1-e^-10^ were further analysed using the BLASTx algorithm against the non-redundant database of NCBI, and those returning the original function were retained.

### Quantitative RT-qPCR analyses

The total RNA was extracted from 0.1 g of the floral tissues with an RNeasy Plant Mini Kit (Qiagen, Hilden, Germany) and treated with DNase-I (Qiagen, Hilden, Germany). Two μg of the RNA was reverse transcribed using oligo(dT)_18_ and the SuperScript III Reverse Transcriptase kit (Life Technologies, Carlsbad, CA, USA), according to the manufacturer’s instructions. After verifying the single amplification products, Quantitative Real-Time PCR experiments were performed using gene-specific primers (S2 Table) for subsets of transcripts (putatively involved in flower development, ovary abortion and pollen-pistil interactions) to verify if their expression profiles corresponded to their putative function. The samples were analysed under the same conditions using three independent biological replicates and three technical replicates. After each assay, the dissociation kinetics were analysed to determine the specificity of the amplified products. The relative amounts of all mRNAs were calculated using the ΔΔCT method. The housekeeping genes *Elongation Factor 1*-α and *Actin-7a* were used as endogenous reference loci to normalize the expression levels of the target genes.

## Results

### Transcriptome sequencing, assembly and functional annotation

Pyrosequencing of four cDNA libraries from flowers of Leccino and Dolce Agogia at different stages of pre-anthesis and Leccino and Frantoio at different stages of anthesis (Fig 1) generated a total of 465,331 reads with an average length of 361 bp (median length: 410 bp, longest read: 1,196 bp) (Table 1). As a first step, we assembled the raw reads from the four libraries (Table 2). More than 78% of the raw sequences were included in the assembly, producing a set of 14,599 contigs with an average length of 804 bp. Approximately 73% of sequences were longer than 500 bp, and 53% of contigs were composed of 2–10 reads. The overall distribution was normal: most of the contigs (35.3%) exhibited low-coverage (6–10 reads), while a very small fraction of them (2.8%) exhibited high-coverage (>100 reads). Approximately 92,000 of the reads remained as singletons (19.8%), with an average length of 345 bp. The BLASTx-based annotation of the contigs and singletons, for a total of 106,598 transcripts, allowed us to match approximately 53% of the unigenes to known functional genes; the remaining 49,898 transcripts did not have a function assigned. Most of the annotated unigenes matched the homologous *Vitis vinifera* sequences (49%), followed by *Populus trichocarpa* (18%) and *Ricinus communis* (8%) (S1A Fig). Redundancy among the annotated transcripts of the sequenced dataset resulted in 30,883 different GI identifiers out of 56,700 total transcripts. Assuming a similar level of redundancy among the remaining non-annotated transcripts (49,898), we could estimate that approximately 58,000 unique transcripts, representing 54.42% of the sequenced dataset (contigs plus singletons), are expressed in *Olea* flower tissues.

**Table 1:** An overview of the raw data from the 454-FLX Titanium sequencing.

Sample	Cultivar	Flower stages	Number of bases	Reads	Mean length (bp)
L1-6	Leccino	1-2-3-4-5-6	42,568,083	113,134	376
L7-10	Leccino	7-8-9-10	21,939,320	67,797	324
DA1-6	Dolce Agogia	1-2-3-4-5-6	54,807,276	146,576	374
F7-10	Frantoio	7-8-9-10	48,459,361	137,824	352
TOTAL			167,774,040	465,331	361

**Table 2:** An overview of the assemblies that putatively referred to flower development (FD), ovary abortion (OA) and pollen-pistil interactions (PPI).

	Contigs				Singletons	
Samples in the Assembly	Reads in the assembly	%	Number	Average length (bp)	Number	Average length (bp)
Total (L1-6, DA1-6, L7-8, F7-8)	338,853	78.65	14,599	804	91,999	345
1. FD (L1-6, L7-8)	109,764	65.12	5,292	733	58,794	351
2. OA (L1-6, DA1-6)	171,774	71.16	8,235	764	69,624	365
3. PPI (L7-8, F7-8)	126,408	65.79	6,155	757	65,745	335

Approximately 1/4 and 1/20 of the unigene set (24.5% and 5.9%, respectively) were assigned to GO and KO terms, respectively (S3 Table). Concerning the GO classification, the unigenes were grouped into 9 categories of cellular components, 14 categories of biological processes and 11 categories of molecular functions (S1B Fig). The metabolic and cellular processes categories, consisting of 11,103 and 10,609 transcripts, respectively, were the dominant biological processes, whereas the cell part category, with 8,226 unigenes, was the most consistently represented cellular component. The binding and catalytic activity categories, with 13,045 and 10,894 singletons/TCs, respectively, were the dominant molecular functions.

The main biological pathways that are active in olive flowers were mapped using the KEGG database and allowed us to assign 6,272 KO genes into 237 KEGG categories (S3 Table). The most frequently represented biological processes/metabolic pathways included biosynthesis of secondary metabolites (282 unigenes), RNA transport (85), oxidative phosphorylation (68), purine metabolism (63), ubiquitin mediated proteolysis (62), endocytosis (40) and glycolysis/gluconeogenesis (39).

### Differential gene expression between cultivars and developmental stages

To identify the differences between cultivars and specific flower developmental stages that may be related to important flower biological processes, such as flower development (FD), ovary abortion (OA) and pollen-pistil interactions (PPI), we constructed three independent assemblies of the raw sequences (259,710). FD included sequences from Leccino flowers at pre-anthesis and anthesis (L1-L6 vs. L7-L10); OA included the data from the low- and high-ovary aborted flowers of Leccino and Dolce Agogia, respectively (L1-L6 vs. DA1-DA6), and PPI encompassed the data from the flower samples of Leccino and Frantoio at anthesis (L7-L10 vs. F7-F10), which are characterized by a contrasting methods of successful self-incompatibility.

Approximately 60%, 66% and 68% of the raw sequences from the FD, OA and PPI data were assembled into 5,292, 8,235 and 6,155 contigs, respectively. All contigs from the three assemblies were mapped on the entire unigene set, and subsequently annotated and functionally classified using the same procedure described above. Nevertheless, the functional role of a number of up- and down-regulated sequences with unassigned identity remains to be investigated.

**Fig 2:**
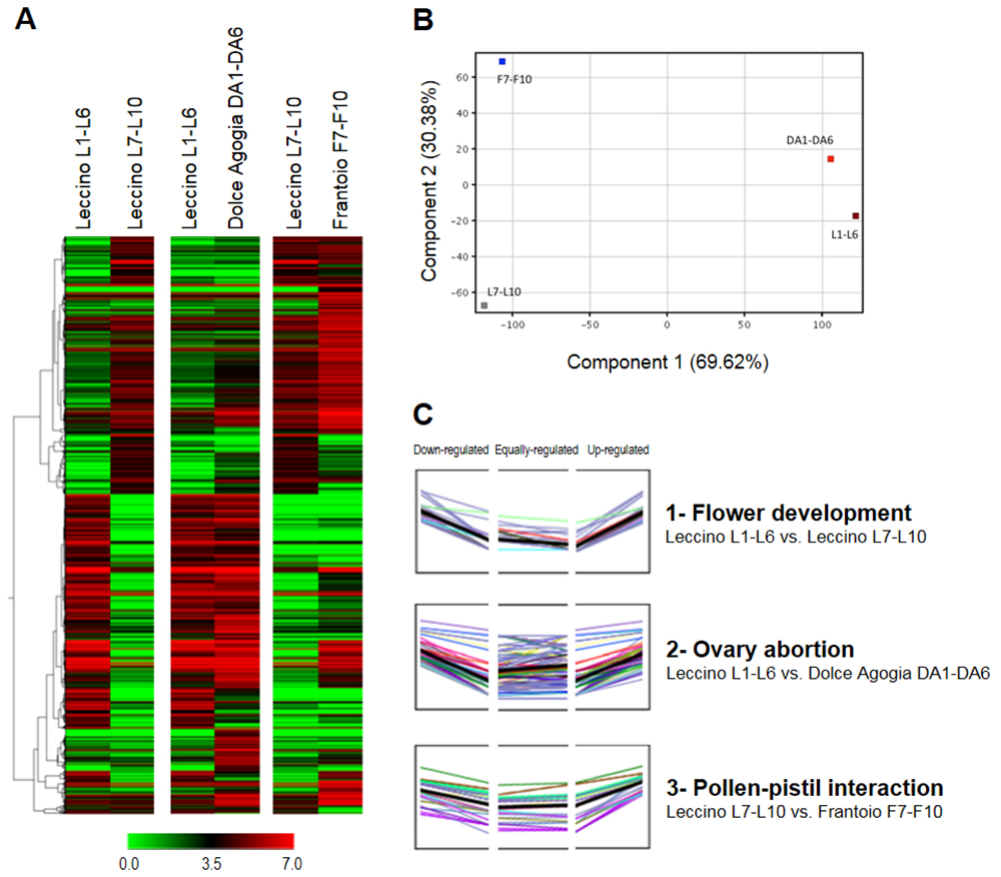
Clustering of the differentially expressed TCs. **(A)** Hierarchical Clustering Analysis (HCA). The different shades of green and red represent the down- and up-regulation levels, respectively. **(B)** Principal Component Analysis (PCA). The percentage of variance explained by each component is shown within brackets. **(C)** Clustering of the co-expressed transcripts. L1-L6 = Leccino at pre-anthesis, DA1-DA6 = Dolce Agogia at pre-anthesis, L7-L10 = Leccino at anthesis, and F7-F10 = Frantoio at anthesis.

To identify the representative expression patterns of the regulated genes, we performed multivariate analyses based on the similarities of gene expression (Fig 2). The Hierarchical Clustering Analysis (HCA) of the differentially expressed TCs distinguished the gene transcripts that were differentially expressed between the different flower developmental stages (L1-L6 vs. L7-L10) from those that were differentially expressed between genotypes for ovary abortion (L1-L6 vs. DA1-DA6) and pollen-pistil interactions (L7-L10 vs. F7-F10), demonstrating that the differences between the flower developmental stages were larger than those between the cultivar genotypes (Fig 2A). Moreover, the comparison of differences in expression between L1-6 and DA1-6 were smaller than those between L7-L10 and F7-F10, as confirmed by Principal Component Analysis (PCA) (Fig 2B). The co-expressed transcripts were clustered, with similar models of up-regulated, equally regulated and down-regulated genes across the different comparisons of the flower stages or plant genotypes (Fig 2C).

**Fig 3:**
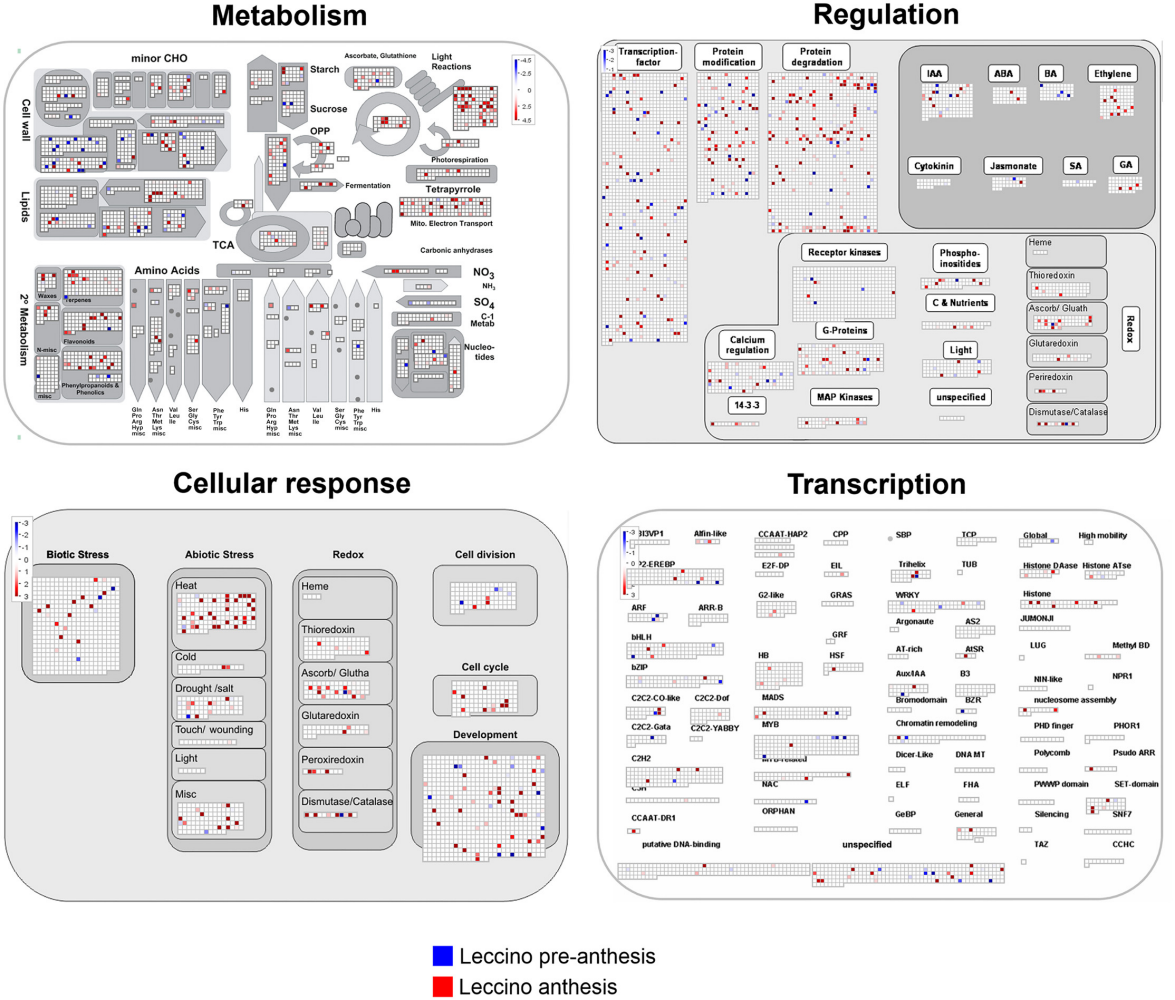
MapMan overview. The MapMan overview maps related to metabolism, regulatory network, cellular response and transcription show differences in the transcript levels between the Leccino flowers at pre-anthesis (stages 1–6) and anthesis (stages 7–10).

The MapMan maps provided an overview of the genes involved in the olive flower metabolism by grouping the sequences into key regulatory networks (Fig 3, S2 and S3 Figs). This analysis enabled us to identify the main pathways modulated in the flower samples under study and putatively related to flower development, ovary abortion and pollen-pistil interactions. The transcriptome data generated from the flower buds collected at stages L1-6 (pre-anthesis) and L7-10 (anthesis) were mapped to identify the transcripts that were expressed during flower development in Leccino (Fig 3). Concerning the flower metabolism, we could annotate and map numerous genes of the primary and secondary metabolism, such as those involved in light reactions, photorespiration and the electron transport chain in mitochondria. Based on the regulatory mechanism, we could identify and map 149 transcription factors that are expressed during flower development. Furthermore, 76 and 178 gene products expressed during stages L1-6 and L7-10, respectively, were mapped in the context of post-translational modifications and protein degradation processes. The large number of genes involved in the modification and degradation of proteins represents the substantial changes that occur during the development of all flower verticils, including reproductive organs. Transcripts involved in hormone metabolism were also present in the flower samples. Although the mapping approach was meant to provide an overview of the data and the mapped values are not statistically significant, it is worth mentioning that the gene products involved in metabolism of ethylene, abscisic acid and gibberellins were primarily identified in the L7-10 samples, which correspond to later developmental stages. A similar trend was observed for the genes involved in the response to biotic stresses (Fig 3).

The same approach was adopted to classify the gene transcripts with known roles in the regulation of gamete and seed formation. Among all of the genes involved in sporogenesis and gametogenesis, *AML4* (unigene00565), *CHR11* (unigene00661), *AtTLP6* (unigene02161), *BRI1* (unigene03435) and *AFB2* (unigene00205), associated with cell division, transcriptional regulation, hormone metabolism and signalling, were identified in the pre-anthesis flowers (Fig 3). The sequence dataset covered mRNAs that are expressed at anthesis and may be associated with gametogenesis (*ATRAB2*, unigene07486; *ATMPK4*, unigene01741, unigene01358) and embryo and seed development (*UNE12*, unigene03070; *MEE34*, unigene06571).

Similarly, the data collected from OA and PPI assemblies were used to depict overview maps of the metabolic, cellular and regulative processes (S1 and S2 Figs). One key finding, that emerged from the analysis of these libraries, was that numerous genes directly involved in sucrose and starch metabolism, cell wall metabolism, lipid metabolism and genes encoding for enzymes of phenylpropanoid and flavonoid pathways were mapped (S2 and S3 Figs).

**Fig 4:**
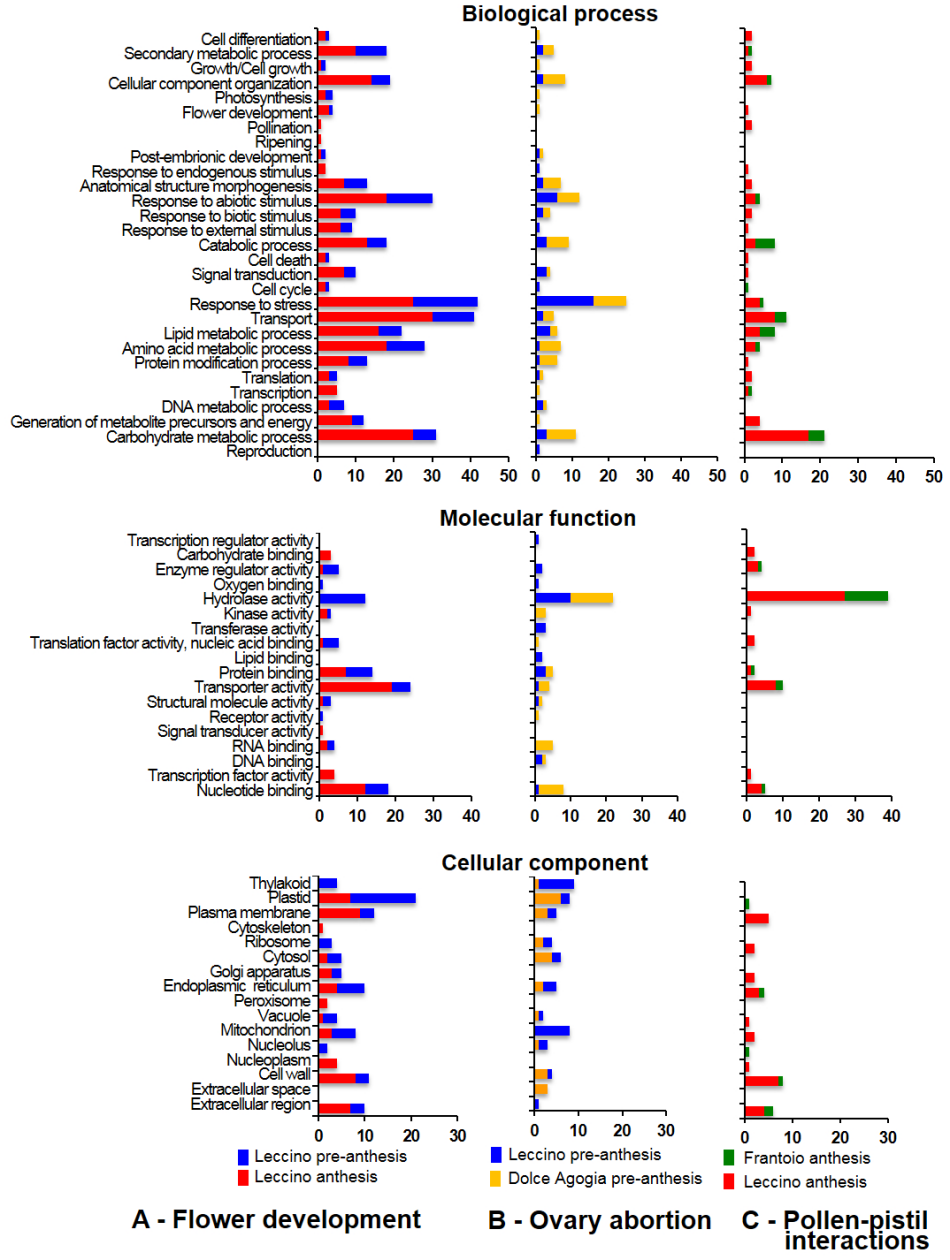
GO terms for biological processes. Distribution of the differentially expressed transcripts related to **A**—flower development, **B**—ovary abortion, and **C**—pollen-pistil interactions in the main GO categories for “biological process”, “molecular function” and “cellular component”.

To reduce the whole sets of differentially expressed genes to a statistically significant subset, the statistical R test [39] was applied to each of the three assemblies to identify genes involved in FD, OA and PPI. All TCs with R>8 (true positive rate of ~98%) were considered differentially expressed. A total of 404 differentially expressed contigs, of which 197, 123, and 84 were present in the FD, OA, and PPI assemblies, respectively (S4, S5 and S6 Tables). The computational analysis of these data using the software BLAST2GO enabled the annotation of the up- and down-regulated expressed sequences according to the terms of the three main Gene Ontology terms: cellular compartment, molecular function and biological process (Fig 4).

Stage-related comparisons between pre-anthesis and anthesis in Leccino provided a general picture of the metabolic pathways that were most affected during flower development (S4 Table, Fig 4A). Many genes involved in carbohydrates (25), lipid metabolism (21), transport (30) and cellular component organization (14) were differentially expressed between pre-anthesis and anthesis, and most of them were up-regulated. Five transcripts encoding for cell wall-remodelling proteins, such as pectinases (unigene01320|FD|contig00279), polygalacturonases (unigene01310|FD|contig00259, unigene01077|FD|contig00195, unigene01547|FD|contig00217,) and expansin (unigene02619|FD|contig00832), were up-regulated at anthesis, as well as a putative S-adenosylmethionine decarboxylase that is involved in polyamine biosynthesis (*OeSAMDC*, unigene00546|FD|contig00142) (S4 Table). The expression of ten genes involved in secondary metabolite biosynthesis was modulated during flower development, eight of which were down-regulated. Among the latter, it is worth mentioning those that are involved in phenylpropanoid and flavonoid biosynthesis, such as chalcone synthase (*OeCHS*, unigene01823|FD|contig00275, unigene01413|FD|contig00302), flavonol synthase (*OeFLS*, unigene02203|FD|contig00893, unigene02203|FD|contig02240) and chalcone isomerase (*OeCHI*, unigene03725|FD|contig01267) (S4 Table).

For the molecular function group, the most represented category among the down-regulated genes at anthesis was hydrolase activity, which was completely absent among the up-regulated genes. In contrast, of the up-regulated genes, the most diffused function was the transporter activity. Most of the transcripts involved in receptor activity, oxygen binding, enzyme regulation or translation factor activities were down-regulated. Finally, in the cellular compartment group, the plastid category was the most represented category in the down-regulated genes at all anthesis stages, whereas the most represented categories of the up-regulated genes were the plasma membrane, plastid, cell wall and extracellular region.

Interestingly, comparing Leccino and Dolce Agogia at pre-anthesis (stages 1–6), emerged that a large number of genes up-regulated in the highly-staminate flowers of Dolce Agogia was associated with cellular component organization, anatomical structure morphogenesis, catabolic process, protein modification and carbohydrate metabolism (Fig 4B), suggesting that these biological processes may be related to ovary abortion. On the other hand, most of the down-regulated genes were involved in the responses to stress and abiotic stimuli. Hydrolase activity was the molecular function category containing the highest number of differentially expressed transcripts (Fig 4B). Kinase, transport, RNA and nucleotide binding functions were also significantly represented among the up-regulated genes, whereas, transferase, protein and lipid binding activities were more represented in the down-regulated genes compared to those up-regulated. Most of the down-regulated genes belonged to mitochondrion and thylakoid cellular components, whereas, the up-regulated genes were mainly associated with the plastid and cytosol categories.

The computational analysis also enabled us to identify a large set of genes putatively involved in pollen-pistil interactions and differentially expressed between Frantoio and Leccino cultivars at anthesis (stages 7–10), when the pollen grains reach the stigma upon self-pollination and potentially trigger the incompatibility reaction (Fig 4C). The most represented biological process categories among the genes that were up-regulated in Leccino (self-incompatible) were carbohydrate metabolic process, transport and cellular component organization. Remarkably, we found that more than 26% of the up-regulated genes in the self-incompatible Leccino were involved in cell wall degradation, whereas ontological terms related to catabolic metabolism were enriched in the up-regulated genes of Frantoio (self-compatible). Most of the differentially expressed genes belong to hydrolase molecular function category. It is worth noting that most of the genes that were over-represented in Leccino are associated with the following cellular component categories: cell wall, plasma membrane and extracellular region.

The transcriptome sequencing of the four libraries generated a repertory of flower genes suitable for the study of flower metabolism. The following computational analyses of differentially expressed genes provided a general overview of the pathways and functional categories modulated between pre-anthesis and anthesis stages and between different cultivars. After these analyses, we performed a more accurate study of the target processes (*i*.*e*., flower development, ovary abortion and pollen-pistil interactions) by searching for homologous genes from other plant species and performing RT-qPCR analyses on 33 candidate genes using single flower tissues and/or developmental stages. This approach enabled us to overcome the limits of the initial sequencing mainly due to the pool of different developmental stages and flower tissues and to the low sequencing depth of each library. Accordingly, important genes related to flower development, ovary abortion and pollen-pistil interactions were identified and their expression profile was analysed.

### Identification of the genes involved in olive flower development

**Fig 5:**
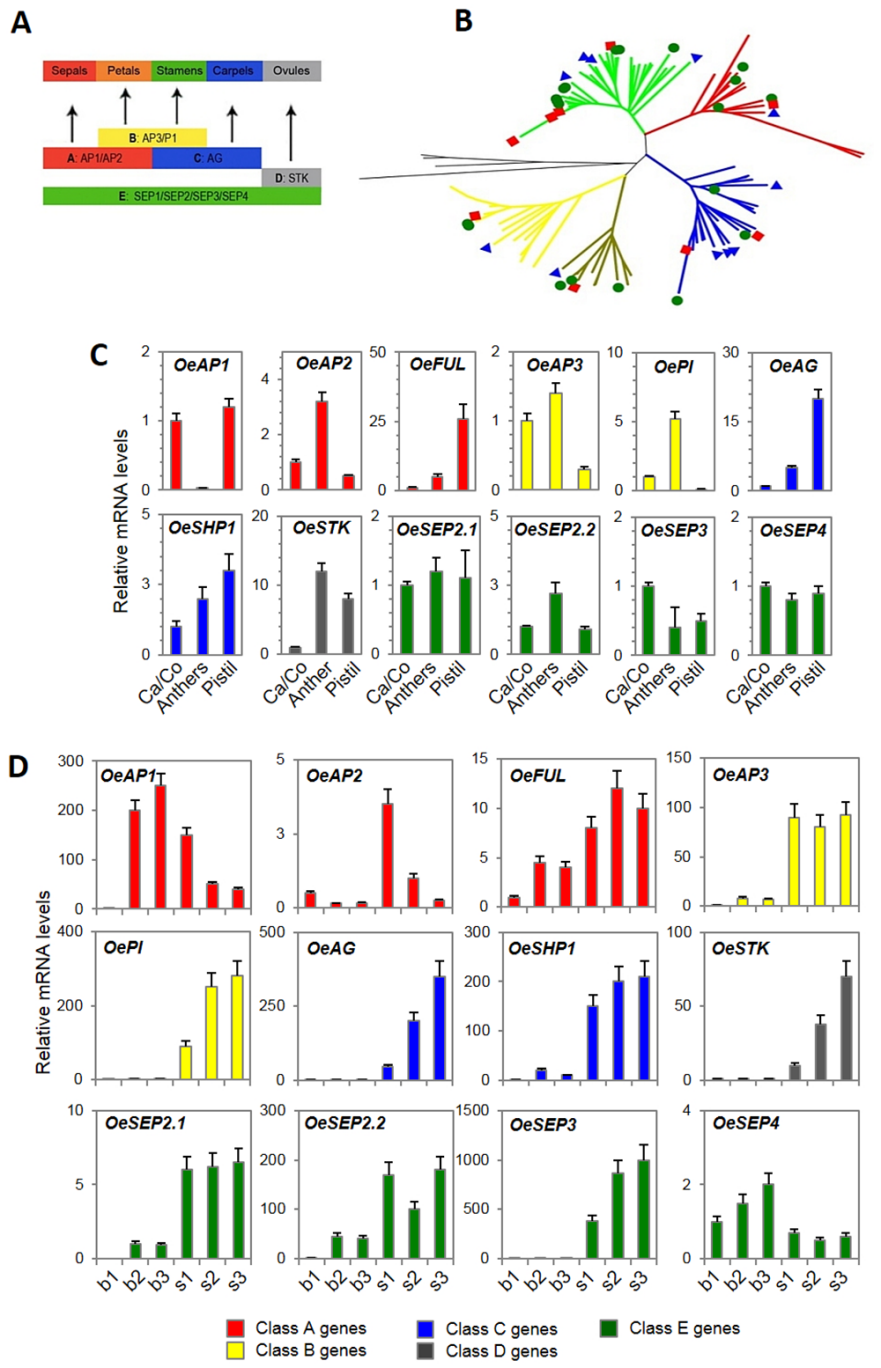
Expression of floral organ identity genes. The genes involved in floral organ identity, according to the ABC model, are expressed in the olive flowers. **(A)** Schematic representation of the classical ABC model for the development of flower organs. Class A genes (red) specify sepals, class A and B (yellow) genes specify petals, class B and C (blue) genes specify stamens, class C genes specify carpels, class D genes (grey) are specifically expressed in ovules, and class E genes (green) have partially redundant functions in floral organ identity determination. **(B)** The un-rooted NJ tree of key determinants of floral organ identity emphasizes the relationships between the different ABCDE-class genes. The five main protein groups that were identified are indicated with different colours: *SEPALLATA* (*SEP*, green), *APETALA1* (*AP1*, red), *AGAMOUS* (*AG*, blue), *PISTILLATA* (*PI*, yellow) and *APETALA3* (*AP3*, brown). The green circles, red rectangles and blue triangles represent the proteins from *Olea europaea*, *Antirrhinum majus* and *Arabidopsis thaliana*, respectively. **(C, D)** Relative mRNA levels of 12 MADS-box genes in single flower parts (Ca/Co: calix and corolla, anther, pistil) of flowers at S4 stage **(C)** or whole inflorescence buds (b1-b3) and differentiated flowers (stages 1–3) at progressive developmental stages **(D)** of Leccino. *OeAP1*: unigene03554; *OeAP2*: unigene00847; *OeFUL*: *FRUITFULL*, unigene01990; *OeAP3*: unigene04520; *OePI*: unigene04760; *OeAG*: unigene03555; *OeSHP1*: *SHATTERPROOF*, unigene03887; *OeSTK*: SEEDSTICK, unigene07062; *OeSEP2*.*1*: unigene04526; *OeSEP2*.*2*: unigene02210; *OeSEP3*: unigene03885; and *OeSEP4*: unigene04898. The data were obtained using RT-qPCR. The values are the means of three biological replicates and the SD is indicated.

We adopted a self-confirming bioinformatics approach to identify the genetic determinants (Table 3) that contribute to the ABC model (Fig 5A) of floral organ identity control in olive. The MADS-box genes, which belong to the MEF2 (myocyte enhancer factor 2)-like/Type II subfamily of MADS (MCM1, AGAMOUS, DEFICIENS and SRF box family) and are mainly involved in regulating the determination of flower organ identity and development, were analysed to shed some light on their composition and expression. By restricting the BLAST analyses to this gene group, we could identify 20 putative olive orthologous of MADS-box genes. The same approach led to the identification of two putative orthologues of APETALA2 (*OeAP2*). As reported in Table 3, the list of identified putative transcripts included the following candidates: two *AP1*, two *AP2* (*APETALA2*) and two *FUL* (*FRUITFULL*) for the class A genes according to ABC model for floral organ identity; two *AP3* and three *PI* for class B; one *AG* and one *SHP1* (*SHATTERPROOF*) for class C; one *STK* (*SEEDSTICK*) for class D; and two *SEP2* (*SEP2*.*1*, *SEP2*.*2*), five *SEP3* and one *SEP4* for class E. The genes belonging to the A, B and E classes were highly redundant, as multiple olive ESTs matched the same gene. As expected, the class E genes, represented by the *SEPALLATA* group, showed the highest degree of redundancy. In accord with taxonomic relationships of the reference species, BLAST searches over the reference databases indicated a high level of conservation between the *Olea* and *Antirrhinum* sequences, and most of the BLAST searches retrieved *A*. *majus* accessions as the best matches.

**Table 3:** Genes involved in ABC model flower.

Gene model	Gene^a^	Arabidopsis orthologs	BLAST best match	EST ID	E-value^b^	Score
**A**	***APETALA1* (*OeAP1*)**	NP_177074.1	[*A*. *majus*] CAA45228.1	unigene03554*	1e^-128^	360
				F7KHMQ102C4L9M	1e^-97^	273
**A**	***APETALA2* (*OeAP2*)**	NP_195410.1	[*A*. *majus*] AAO52747.1	unigene00847*	2e^-124^	369
				unigene03848	4e^-67^	206
**A**	***FRUITFULL* (*OeFUL*)**	NP_568929.1	[*A*. *majus*] AAK72467.1	unigene01990*	4e^-117^	330
				unigene03352	5e^-108^	304
***B***	***APETALA3* (*OeAP3*)**	NP_191002.1	[*A*. *majus*] P23706.1	unigene04520*	2e^-153^	420
				unigene04701	2e^-145^	420
***B***	***PISTILLATA* (*OePI*)**	NP_197524.1	[*A*. *majus*] Q03378.1	unigene04760*	3e^-81^	237
				unigene04116	8e^-125^	369
				unigene05763	4e^-74^	237
***C***	***AGAMOUS* (*OeAG*)**	NP_567569.3	[*A*. *majus*] CAB42988.1	unigene03555*	2e^-144^	403
***C***	***SHATTERPROOF (OeSHP1)***	NP_191437.1	[*A*. *thaliana*] AAB25101.1	unigene03887*	4e^-101^	285
***D***	***SEEDSTICK* (*OeSTK*)**	NP_192734.1	[*Petunia x hybrida*] CAA57311	unigene07062*	3e^-85^	244
***E***	***SEPALLATA2* (*OeSEP2*)**	NP_186880.1	[*A*. *majus*] CAA64741.1	unigene04526*	2e^-150^	414
				unigene02210	1e^-141^	416
***E***	***SEPALLATA3* (*OeSEP3*)**	NP_564214.2	[*A*. *majus*] CAA64743.1	unigene03885*	1e^-115^	328
				unigene03704	6e^-135^	396
				unigene03748	8e^-135^	395
				unigene03982	3e^-136^	399
				unigene04216	4e^-146^	424
***E***	***SEPALLATA4* (*OeSEP4*)**	NP_178466.1	[*Petunia x hybrida*] AAK21247.1	unigene04898*	7e^-95^	291

Olive genes encoding key proteins of flower development, according to the ABC model. The genes were confirmed by RT-qPCR experiments.

^a^The nomenclature from the *Arabidopsis thaliana* orthologs has been used.

^b^E-value of the best hits identified by a BLASTX search.

*Transcripts selected for the RT-qPCR analyses.

The alignment of the deduced protein sequences from olive with a comprehensive set of proteins (S1 Table) that have previously been characterized in other plant species, such as thale cress, snapdragon, poplar, petunia and tomato, and the following Neighbour-Joining tree analysis, further confirmed that the olive dataset contained at least one representative for each of the considered gene models (Fig 5B). It was possible to identify the presence of five main protein groups (Fig 5B), which were significantly differentiated from each other (bootstrap values greater than 99%) and named according to the Arabidopsis nomenclature, such as SEPALLATA (SEP), APETALA1 (AP1), AGAMOUS (AG), PISTILLATA (PI) and APETALA3 (AP3). In a more detailed phylogenetic tree (S4 Fig), the SEEDSTICK (STK) proteins grouped together with AG, and a distinct subgroup was identified (minimum bootstrap value of 95%).

According to the overall accepted ABC model of flower development (Fig 5A), the expected expression pattern of the class A genes should be in calix and corolla, the class B genes should be expressed in the corolla and stamens, the class C genes should be expressed in the stamens and carpels, and the class D genes should be expressed in the ovules. However, the class E genes, which have partially redundant functions, might be expressed in different floral organs.

A number of RT-qPCR experiments (Fig 5C) were exploited to confirm the annotation of olive MADS-box genes and to further investigate whether the generally accepted expression pattern of the flower identity genes is conserved in this species. The results showed that the class A gene *OeAP1* was detected in the calyx, corolla and pistil, whereas, unexpectedly, higher expression levels of *OeACP2* and *OeFUL* were observed in anthers and pistils, respectively. In accord with their function, the class B genes *OeAP3* and *OePI* were primarily expressed in the calix/corolla and anthers, whereas the expression of the class C gene *OeAG* was higher in the pistils. The expression pattern of *OeSHP1* was similar to the related *OeAG* gene, but at a lower level, with non-significant differences in the expression between whorls. The class D *OeSTK* transcripts, which would only be expected to be expressed in the most internal whorls, were clearly detectable in both the anthers and pistils. For the class E genes, four distinct *OeSEP* members were equally expressed within the different flower parts, with the exception of *OeSEP2*.*2*, which was slightly over-expressed in the anthers.

For the same set of genes, differential expression was also evident during inflorescence and flower development (Fig 5D). Based on the presence of the transcripts in the inflorescence buds and flowers, the analysed genes could be grouped as early-expressed genes, whose transcripts were already detectable in the inflorescence buds, and late-expressed genes, which were only observed in single flowers. A first group contains *OeAP1* and *OeSEP4*, as both genes reached their highest expression level at late stages of the inflorescence bud. In contrast, a second group of genes, which includes *OeAP3*, *OePI*, *OeAG*, *OeSHP1*, *OeSTK* and *OeSEP3*, were exclusively expressed in single flower samples, suggesting a role in later developmental stages. A third group is represented by the genes that exhibited low expression levels at early developmental stages and then increased at later developmental stages in both the inflorescence buds and flowers. This group of genes is ideally composed of the olive orthologues of *OeAP2*, *OeSEP2* and *OeFUL*. Finally, the expression of *SEP4* was not significantly change during flower development, with slightly higher expression levels in the flower buds.

It is worth mentioning that class A genes, such as *OeAP1*, displayed an expression pattern that was opposite that of the C and D class genes *OeAG* and *OeSTK*.

### Identification of the genes that may be involved in ovary abortion

The main results of the BLAST-based analyses, used to identify the genes that may have a role in ovary abortion, are summarized in Table 4. The olive homologues of genes involved in starch and sucrose metabolism, polyamine biosynthesis, cell wall metabolism, programmed cell-death (PCD), regulation of flavonoid biosynthesis, and MYB and MADS transcription factors were identified.

**Table 4:** Genes involved in ovary abortion.

Gene	Biological Process	References	BLAST best match^a^	EST ID^b^	Length (bp)	E-value^c^
***β-amylase* (*BAM1*)**	Starch and sucrose metabolism	[42, 43]	[*N*. *tabacum*] AAY89374.1	unigene01162*	1,600	0.0
				unigene10049	284	1e^-132^
				unigene13639	280	1e^-126^
				F7KHMQ102EZ5BY	147	1e^-34^
				F7KHMQ102EYM72	131	8e^-30^
				F7KHMQ102D1LWV	117	1e^-25^
***Cell-wall invertase* (*Inv-CW*)**	Starch and sucrose metabolism	[44, 45]	[*O*. *ramosa*] ADF27781.1	unigene02494*	879	1e^-166^
***Soluble acid invertase 1* (*Inv-V*)**	Starch and sucrose metabolism	[45, 46]	[*O*. *ramosa*] ADF27779.1	unigene01665*	1,283	1e^-151^
				F7KHMQ102DGVGZ	478	6e^-40^
***Granule bound starch synthase I* (*GBSSI*)**	Starch and sucrose metabolism	[47]	[*N*. *tabacum*] AGH30272.1	unigene00185*	1,834	0.0
				F7KHMQ102C5WN5	225	3e^-18^
***Sucrose synthase* (*SuSy*)**	Starch and sucrose metabolism	[48]	[*S*. *lycopersicum*] AAO67719.1	unigene02089*	1,311	0.0
				unigene00331	971	0.0
				unigene04915	937	8e^-161^
				unigene03893	896	2e^-144^
***N-carbamoylputrescine amidase* (*NLP1*)**	Polyamine biosynthesis	[49]	[*A*. *thaliana*] Q8VYF5.1	unigene03141*	1,103	0.0
***Spermidine synthase* (*SPDS*)**	Polyamine biosynthesis	[50]	[*O*. *europaea*] ACZ73829.1	unigene01420*	1,424	0.0
				unigene01406	1,105	0.0
				unigene01897	716	1e^-151^
***Spermine synthase* (*SPMS*)**	Polyamine biosynthesis	[51]	[*A*. *thaliana*] NP_851178.1	unigene06009*	1,223	0.0
				F7KHMQ101B5AWP	496	1e^-65^
***Arginine decarboxylase* (*ADC*)**	Polyamine biosynthesis	[52]	[*S*. *lycopersicum*] CAI39242.1	unigene00039*	3,02	0.0
				unigene05970	744	1e^-163^
				F7KHMQ104H5U8P	323	3e^-87^
				F7KHMQ102EIJ8S	270	3e^-71^
***Transcription factor* (*MYBPA1*)**	Flavonoid pathway	[53]	[*V*. *vinifera*] CAJ90831	unigene0379	100	5e^-6^
				F7KHMQ104JBLBN	504	9e^-45^
				F7KHMQ102DYFKF	491	1e^-27^
				F7KHMQ102DY898	313	2e^-22^
***Transcription repressor MYB* (*MYBC2*)**	Flavonoid pathway	[54]	[*V*. *vinifera*] XP_002278222.1	unigene04022*	981	1e^-91^
				F7KHMQ104H6D9V	93	2e^-17^

Olive candidate genes putatively involved in ovary abortion. The genes were confirmed by RT-qPCR experiments.

^a^Species and accession number of the genes that were used as reference sequences for the tBLASTx search.

^b^Best hits of the olive transcripts, as identified using a bi-directional BLAST search.

^c^E-value of the best hit identified by the BLASTX search.

*Transcripts selected for the RT-qPCR analyses.

**Fig 6:**
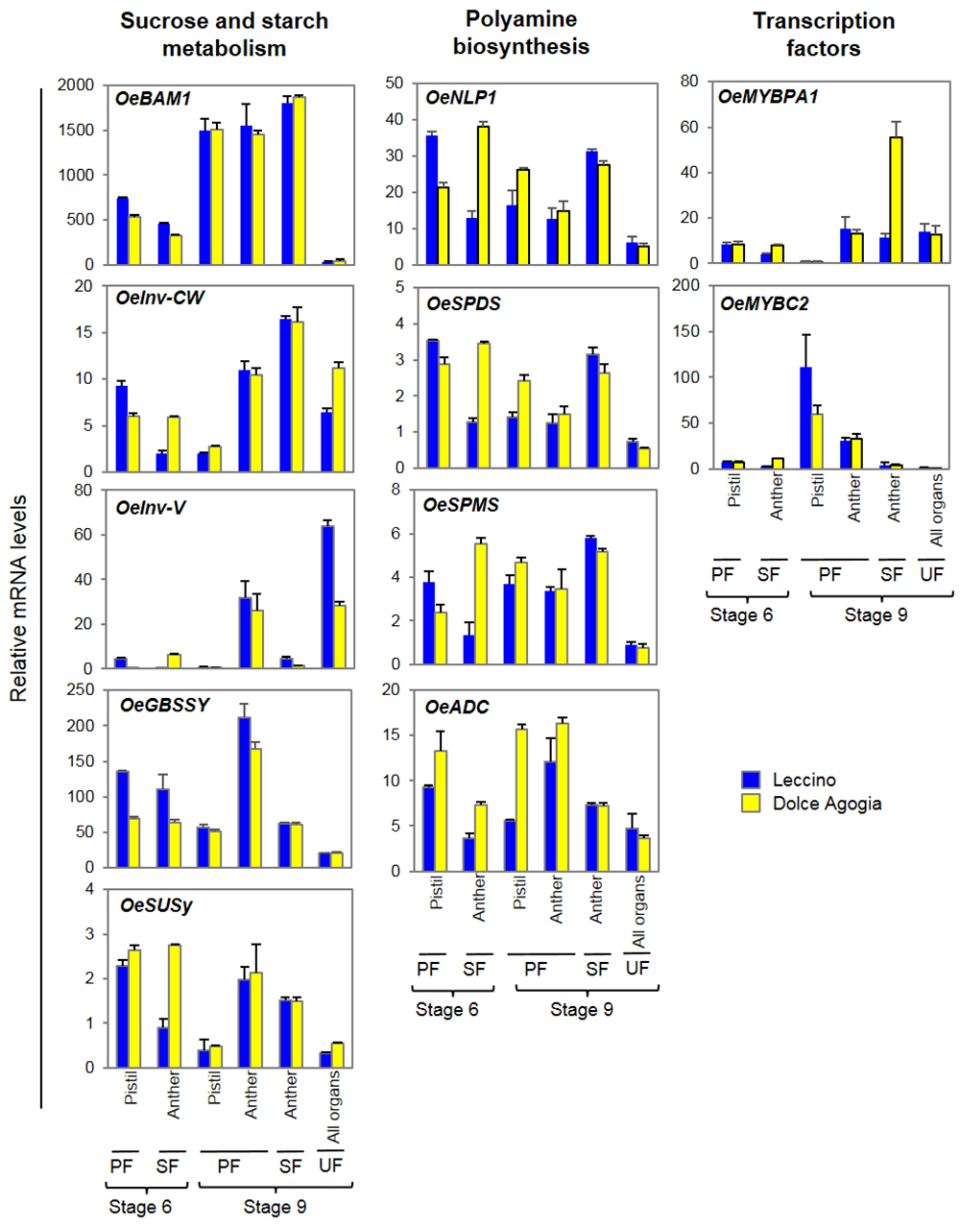
Expression of ovary abortion genes. Relative mRNA levels of the genes potentially related to ovary abortion, as determined by RT-qPCR. Flowers at stages 6 and 9 were analysed. *OeGBSSI*: Granule bound starch synthase I, unigene00185; *OeSuSy*: sucrose synthase, unigene02089; *OeBAM1*: β-amylase1, unigene01162; *OeInv-CW*: cell-wall invertase, unigene02494; *OeInv-V*: soluble acid invertase, unigene01665; *OeADC*: Arginine decarboxylase, unigene00039; *OeNLP1*: N-carbamoylputrescine amidase, unigene03141; *OeSPDS*: Spermidine synthase, unigene01406; *OeSPMS*: Spermine synthase, unigene06009; *OeMYBPA1*: unigene03795; and *OeMYBC2*: unigene04022. The transcripts were detected in pistils and anthers of perfect flowers, anthers of staminate flowers and entire undeveloped flowers. The values are means of three biological replicates and the SD is indicated.

The differential expression of the most interesting genes was validated by RT-qPCR (Fig 6) of pistils and anthers from both perfect (PF) and staminate flowers (SF) of Leccino and Dolce Agogia at two developmental stages: stage 6 and stage 9 (Fig 1). Moreover, whole undeveloped flowers (UF) at stage 9 were also used in the analysis.

Among the genes involved in starch and sucrose metabolism, a putative β-amylase (*OeBAM1*), a cell-wall invertase (*OeInv*-*CW*), a vacuolar soluble acid invertase (*OeInv-V*), a granule bound starch synthase-I (*OeGBSSI*), and a sucrose synthase (*OeSuSy*) were analysed (Fig 6). We observed differences in their mRNA levels that might affect the correct balance of nutrients and starch accumulation in olive flower organs in accordance to their putative role in ovary abortion.

*OeBAM1* was highly expressed in both anthers and pistils at stage 9 compared to stage 6 in both genotypes, and was almost not expressed in the undeveloped flowers. *OeInv-CW* and *OeInv-V* were poorly expressed at the first stages of development compared to the full anthesis in both perfect and staminate flowers; however, the mRNA levels of *OeInv-CW* in the anthers of staminate flowers were significantly higher compared to the anthers of perfect flowers, whereas, the expression of *OeInv-V* was definitely lower. Moreover, the *OeInv-V* mRNA levels were lower in the pistils of Dolce Agogia compared to Leccino. The RT-qPCR analysis revealed that the *OeGBSSI* transcripts, which encode an enzyme that catalyses amylose synthesis, were present in both floral whorls, with the highest expression in the anthers of the perfect flowers at the late stage, as observed for *OeInv-V*. The most visible changes in *OeSuSy* mRNA expression were observed in the pistils of perfect flowers, and significantly decreased throughout flower development.

Among the genes involved in polyamine biosynthesis, we analysed by RT-qPCR N-carbamoylputrescine amidase (*OeNLP1*), spermidine synthase (*OeSPDS*) spermine synthase (*OeSPMS*) and arginine decarboxylase (*OeADC*) (Fig 6). Two of them (*OeADC* and *OeSPDS*) resulted previously differentially expressed, applying statistical R test, between entire flowers of Leccino and Dolce Agogia at pre-anthesis (S5 Table). RT-qPCR analysis on single flower tissues and developmental stages revealed that the mRNA levels of all of these genes were increased in Dolce Agogia compared to Leccino, confirming the preliminary data on differential expression profile obtained by computational analyses. In addition, the analysis revealed that the strongest differences between genotypes were observed in the anthers of male flowers at stage 6 (primarily *OeNLP1*, *OeSPDS* and *OeSPMS*) and in the pistils of mature flowers at stage 9 (*OeADC* and *OeSPDS*). Interestingly, the expression of three out of the four polyamine genes was reduced in the anthers of perfect flowers compared to staminate flowers. All the polyamine genes were expressed at low levels in the undeveloped flowers. These results indicate that reduced PA levels might be associated with incomplete or abnormal floral organs.

Furthermore, the *OeMYBPA1* and *OeMYBC2* genes, members of R2R3-MYB gene family that encode putative transcription factors and are involved in flavonoid biosynthesis, were also analysed by RT-qPCR (Fig 6). Interestingly, *OeMYBPA1* expression was significantly induced in the anthers of the staminate flowers of Dolce Agogia. The *OeMYBC2* transcripts levels remained quite low in all samples, with the exception of the pistils of the open perfect flowers from both genotypes.

### Identification of the genes that may be involved in pollen-pistil interactions

The candidate gene approach allowed us to expand the set of genes putatively involved in pollen-pistil interactions. Candidates were identified in our libraries based on the sequence similarity to genes characterized in other plant species (Table 5). Homologues of both partners for pollen-pistil interactions were identified, including some lipid transfer proteins (*LTP*), a cysteine-rich extracellular protein (*LAT52*) that interacts with a cysteine-rich protein kinase of the stigma (*PRK1*), a member of the NTP303 family proteins (*NTP*), two pectin methylesterases (*PME*), an oleosin (*OLE*) involved in pollen hydration, and a β-1,3-glucanase (*βGLU*), involved in callose degradation during the process of microspore tetrad dissolution.

**Table 5:** Genes involved in pollen-pistil interaction.

Gene	Biological Process	Tissue	References	BLAST best match^a^	EST ID^b^	Length (bp)	E-value^c^
***Lipid transfer protein precursor* (*LTP*)**	Adhesion of pollen tubes to transmitting tissue	Pistil	[26, 55]	[*L*. *longiflorum*] AF171094	unigene05516	841	3e^-26^
					unigene08514*	610	6e^-33^
					unigene07617*	669	1e^-30^
					unigene07897*	648	1e^-30^
					unigene07066*	708	6e^-29^
***Pollen-specific gene* (*LAT52*)**	Hydration and pollen tube growth	Pollen	[26, 56]	[*S*. *lycopersicum*] CAA33854.1	unigene06374*	764	2e^-53^ 2e^-051^ 1e^-028^ 5e^-028^ 8e^-028^
					unigene06415	762	2e-^051^
					unigene07787	658	1e-^028^
					unigene06119	785	5e-^028^
					unigene05934	802	8e-^028^
					unigene05528	829	9e^-028^
					unigene05190	862	8e^-027^
					unigene06002	797	9e^-027^
					unigene05771	816	2e^-026^
					unigene06255	777	2e^-026^
					unigene05964	800	5e^-026^
					unigene05896	810	8e^-026^
					unigene06066	794	1e^-025^
					unigene07219	684	3e^-024^
					unigene06532	752	3e^-023^
***Pollen-specific gene* (*NTP101*)**	Pollen tube growth	Pollen	[57]	[*N*. *tabacum*] AAQ90182.1	unigene03925*	991	1e^-127^
***Pectin methylesterase* (*PME*)**	Pollen tube growth	Pollen	[58]	[*N*.*acum*] AAX13972.1	unigene00539	1,811	1e^-170^
					unigene00473	1,867	1e^-169^
					unigene01185*	1,493	6e^-041^
					unigene01763*	1,317	5e^-039^
***Oleosin* (*OLE*)**	Pollen hydration	Pollen	[26]	[*C*. *oleifera*] ABF57563.1	unigene08062*	638	3e^-31^
***Beta-1*,*3glucanase* (*βGLU*)**	Callose degradation in the process of tetrad dissolution	Anther	[59]	[*O*. *sativa*] BAC02926.1	unigene00872*	1,632	3e^-056^

Olive candidate genes that are putatively involved in pollen-pistil interactions. The genes were confirmed by RT-qPCR experiments.

^a^Species and accession number of the genes that were used as reference sequences for tBLASTx search.

^b^Best hits of the olive transcripts, as identified by a bi-directional BLAST search.

^c^E-value of the best hit identified by the BLASTX search.

*Transcripts selected for the RT-qPCR analyses.

**Fig 7:**
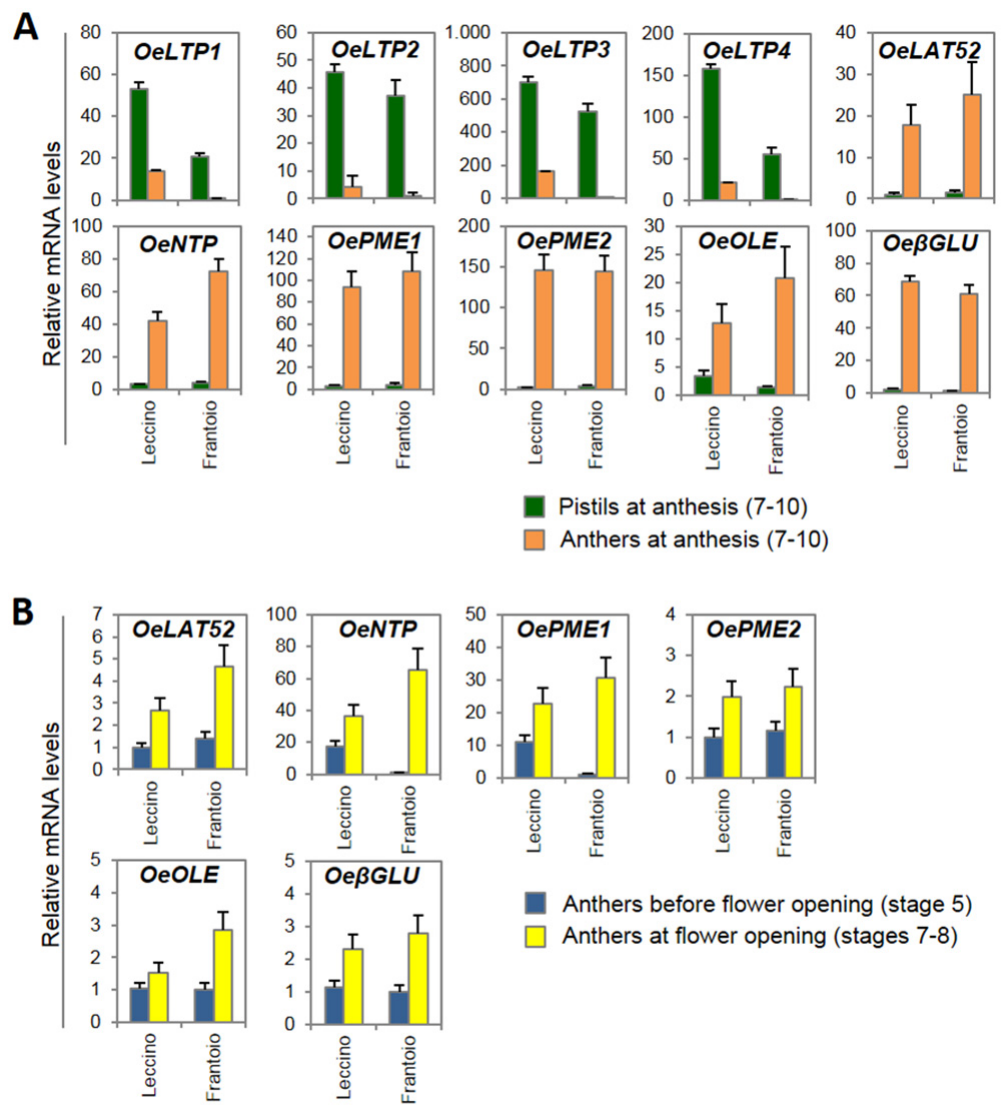
Expression of pollen-pistil interaction genes. Relative mRNA levels, determined by RT-qPCR, of the genes that may be involved in pollen-pistil interactions, **(A)** in anthers and pistils at anthesis (stages 7–10), **(B)** in anthers before anthesis (stage 5) and at anthesis (stages 7–8), from flowers of Leccino and Frantoio. Transcripts analysed include: Lipid transfer proteins (*OeLTP1*: unigene05516; *OeLTP2*: unigene08514; *OeLTP3*: unigene07617; *OeLTP4*: unigene07897, unigene07066); pollen-specific gene (*OeLAT52*: unigene06374); pollen-specific gene (*OeNTP*: unigene03925); Pectin methylesterase (*OePME1*: unigene01185; *OePME2*: unigene01763). Oleosin (*OeOLE*: unigene08062); and β-1,3-glucanase (*OeβGLU*: unigene00872). The values are the means of three biological replicates; the SD is indicated.

The expression levels and patterns of these genes were investigated by RT-qPCR in the anthers and pistils of flowers collected from cvs. Leccino and Frantoio at anthesis (stages 7–10), when the pollen-pistil components should interact (Fig 7A). The results demonstrated that *OeLTP1*, *OeLTP2*, *OeLTP3* and *OeLTP4* possess a pistil-specific expression pattern, whereas the mRNA levels of *OeLAT52*, *OeNTP*, *OePME1*, *OePME2*, *OeOLE* and *OeβGLU* were much higher in the anthers compared to the pistils, in accordance to their function.

The temporal expression of the anther-specific genes was also investigated in the anthers of both cultivars at pre-anthesis (stage 5) and at anthesis (stages 7–8) (Fig 7B). In accordance to their involvement in pollen-pistil interaction, the relative mRNA levels of these genes increased in both cultivars at anthesis, just before the interaction between the pollen-pistil components that will determine the fate of self-pollination process, particularly for *OeNPT* and *OePME1* transcripts in the anthers of Frantoio cultivar.

By preliminary computational analyses, *OePME1*, *OePME2*, and *OeβGLU* resulted differentially expressed in FD (S4 Table) and PPI (S6 Table) datasets of entire flowers. The RT-qPCR analysis confirmed the differential expression between the developmental stages (Fig 7B) but not between the genotypes (Fig 7A) that were not significant.

The putative genetic determinants of self-incompatibility (SI) were also identified (S7 Table), including numerous transcripts of genes that putatively encode the S receptor kinase (*SRK*) and the S-locus glycoprotein (*SLG*) related to sporophytic SI of *Brassicaceae* and a single transcript that is similar to the pistil S-determinant of gametophytic SI of Papaveraceae (*PrsS*). Moreover, the putative SI signalling genes included the M-locus protein kinase (*MLPK*) and the exocyst subunit *EXO70* of the Brassicaceae SI system.

## Discussion

### Sequencing and functional annotation

This study provides a robust portfolio of transcripts expressed in the olive flowers based on distinct developmental stages, reproductive constraints and varietal genotypes. Our data also provide an unprecedented detail on the expression of candidate genes during flower development, ovary abortion and pollen-pistil interaction processes.

Although the transcriptome assembly can present some fragmentation and an under-representation of poorly expressed genes, also because of the lack of a reference genome sequence for the species, we can assume that this study provides a reasonably complete catalogue of the genes expressed in the olive flower, in general, and particularly in the reproductive organs.

### Transcripts that are putatively involved in flower development

A substantial number of genes were differentially expressed between the different flower developmental stages during pre-anthesis and anthesis in Leccino flowers (L1-L6 vs. L7-L10), which may underlie the complex physiological changes that occur throughout the maturation of the reproductive organs. From a functional point of view, the flower could be primarily associated with the development and proper mating of female and male gametes. Hence, a number of genes associated with the main components of gamete formation, including sporogenesis and gametogenesis, were identified.

Various gene transcripts associated with first stages of flower development were related to cell division, regulation of transcription, hormone metabolism and signalling. Major components of the primary and secondary metabolic pathways were significantly modulated during the transition from pre-anthesis to anthesis, and the developing flowers expressed a number of genes involved in ethylene, abscisic acid and gibberellin biosynthesis, signal transduction and responses. The differential expression of mediators that respond to ethylene and gibberellins supports the finding that an ethylene-GA signalling pathway, which is known to regulate plant life cycle progression in response to environmental stimuli [60], might also be a key element in both early and late events of flower development in olive. In accord with the developmental stages considered in this study, the genes related to embryo and seed development were more abundant at later stages, from anthesis to anther dehiscence.

At the onset of anthesis, olive pistils are characterized by a high metabolic activity of the stigmatic papillae and the surrounding tissues, which accumulate starch and lipid materials, as well as pectins, arabinogalactan proteins and many other components to integrate the stigma tissues and the stigma exudate and cuticle [14, 61]. The extracellular components of the olive stigmatic receptive surface appear to participate in the pollen recognition and hydration processes [62]. Hence, the unigenes mapped to the main biological and metabolic pathways that are involved in transport, cellular organization and the responses to various stresses and external stimuli.

Lipid accumulation is a hallmark of pistil maturation in most plant species with wet stigmas. In Solanaceae species such as tomato, tobacco and petunia, the amount of lipid-rich exudates peaks during anthesis and these exudates are critical for controlled pollen hydration [63, 64]. Lipid substances have also been found in the pistil phloem of olive [14] and other plants [65], and may be a consequence of the long-distance transport of lipids. Based on this intense metabolic activity, a significant number of transcripts related to lipid and carbohydrate metabolism were up-regulated in late flower development stages.

Among the other categories of transcripts that were differentially expressed during flower development, numerous transcripts encoding cell wall-remodelling proteins, such as pectinases and polygalacturonases, were up-regulated at anthesis. Both of these classes of proteins presumably act to depolymerize pectins in the primary cell wall and the middle lamella, a structure that pollen tubes need to penetrate in the stylar tissue [66]. Therefore, in mature pistils, these enzymes may be important for degrading the primary wall of the transmitting tract cells or the pectin component of the intercellular material to facilitate the pollen tube growth, as previously hypothesized by other authors [63, 67]. Moreover, two transcripts encoding expansin precursors, which are related to cell wall loosening, were up-regulated, which is in accord with their putative role in fertilization. In fact, it has been previously proposed that pollen and stylar expansin promote pollen tube penetration into the style and pollen tube growth through the transmitting tract [68, 69].

Flavonoids are responsible for flower colour and it has been well established that their content varies substantially throughout flower development and is associated with the modulation of genes involved in their biosynthesis and degradation [70]. Our data indicated that the Oe*CHS*, Oe*FLS* and Oe*CHI* genes are expressed at high levels in the immature flower buds, and their expression then decreases during flower development. However, in *Rhododendron simsii*, other genes in the pathway have shown different temporal expression patterns during flower development [71].

Key proteins in flower development according to the ABC model, including MADS-box genes, a class of transcription factors that are primarily involved in the organogenesis of the flower components, were sequenced and annotated. Detailed investigations aimed at elucidating and verifying the expression pattern of these genes indicated variations in the mRNA abundance in the different flower parts and developmental stages that are largely in agreement with the specific function of each gene in relation to its specific class. The exceptions included the class A genes *OeAP2* and *OeFUL*, whose mRNA levels were higher in anthers and pistils, respectively, compared to the other tissues. According to our data, comparative studies on the ABC model of floral development have revealed lower functional conservation within class A genes compared to the other classes, demonstrating that orthologous genes might have diverse effects on flower morphology in different plant species [72].

It is accepted that the ABC model is conserved among species, such as Arabidopsis, snapdragon, apple, grape, peach, maize and rice, regardless of their different evolutionary histories [70, 6, 73, 74, 75, 76, 77]. Moreover, taking into account the results described in this study, the model is also confirmed in *Olea europaea*. The sequence comparisons highlighted several duplication events that were almost exclusively present in the A- and B-function genes. The functional significance of these duplications remains unknown.

### Transcripts that are putatively involved in ovary abortion

A set of genes that is putatively involved in ovary abortion has been identified by both differential expression analyses of the transcripts between entire flowers of Leccino and Dolce Agogia and by RT-qPCR of the candidate genes in flower organs of perfect and staminate flowers from both cultivars. They include genes related to starch and sucrose metabolism, polyamine biosynthesis, cell wall metabolism, programmed cell-death (PCD), regulation of flavonoid biosynthesis, and MYB and MADS-box transcription factors.

It is well established that starch and sucrose metabolism plays a prominent role in many biological processes of flower development, including flowering time, organ growth [78] and it has also been proposed to have an effect in ovary abortion of olive flowers [10]. Accordingly, we studied the expression profile of some of the key genes of the pathway. *BAM1* encodes an enzyme that catalyses the hydrolysis of starch into maltose [79, 80]. *Inv-CW* and *Inv-V* both encode for invertases, the first localized in the apoplast and latter in the vacuole, that cleave sucrose into glucose and fructose that are directly transported into the sink cells by hexose transporters [81, 82]. *GBSSI* encodes an enzyme that catalyses the amylose synthesis [83]. *SuSy* encodes a sucrose synthase, a key enzyme in the regulation of sucrose accumulation and partitioning [84]. These genes were differentially expressed in the anthers of perfect and staminate flowers, in the pistils at different phased of flower development, and, in some cases, in the cultivars characterized by a different rate of ovary abortion or incomplete differentiation of gynoecium, such as Leccino and Dolce Agogia [85]. Our results indicate that changes in starch synthesis, degradation and mobilization might affect the correct balance of nutrients in flower organs, with consequences on the regular development of pistils and ovary, as previously proposed [10, 86]. It is not known how these processes affect ovary abortion; however, it is clear that flowers need carbohydrates to complete their differentiation, and nutrient deficiency can affect this process by reducing flower organ growth or inducing flower abscission [87, 88]. The down-regulation of cell wall invertases in rice, wheat and tomato affect the sugar content, resulting in alterations in flower and fruit morphology and the rate of pollen tube elongation [89, 90, 44]. The simultaneous down-regulation of *OeInv-CW*, *OeInv-V*, *OeGBSSI* and *OeSUSy* and an up-regulation of *OeBAM1* in the pistils of mature perfect flowers and a strong induction of *OeBAM1* and *OeInv-CW* expression in the anthers of staminate flowers, might indicate that sucrose and starch synthesis and the unloading of sucrose into the sink tissues are less active after anthesis. In contrast, the degradation of the starch produced before anthesis increases, likely to provide energy in preparation of the development of the new organs that will be formed after pollination. The increased expression of genes such as *OeBAM1* and *OeInv-CW* in the anthers of staminate flowers may be related to the role played by these organs to produce as much pollen as possible, which requires carbohydrates as an immediate energy source.

We also identified numerous genes that are involved in polyamine (PA) biosynthesis. In plants, PAs are required for cell growth and division and are implicated in a wide range of biological processes, including floral induction and differentiation, fertility regulation and fruit development [91, 92, 93]; however, their precise roles still remain unclear. The recent discovery that the silencing of the S-adenosylmethionine decarboxylase (SAMDC), which is involved in the PA biosynthetic pathway in the tapetal tissue of tomato flowers, results in male sterility [94], underlines the importance of SAMDC and PAs in pollen development as well. A role for polyamines in male flower sterility/fertility was also hypothesized in different plant species, as sterile lines contain lower PA levels compared to fertile lines [95, 96, 97]. Furthermore, quantitative and qualitative changes in the PA content occur during floral development [91].

In olive, it has been demonstrated that *SAMDC* and *SPDS* transcripts involved in PA biosynthesis are highly regulated in a developmental stage- and tissue-specific manner during anthesis and are related to fruit size and shape [50, 93]. The up-regulation of *OeNLP1*, *OeSPDS*, *OeSPMS* and *OeADC*, in Dolce Agogia anthers of male flowers at an early stage of development, compared to Leccino, may indicate that polyamine metabolism may determine the arrest of pistil development in a significant percentage of flowers. Their lower expression levels in the pistils of perfect flowers may confirm this hypothesis. The evidence that undeveloped olive flowers with aborted ovaries and undeveloped stamens contain reduced mRNA levels of the four PA genes analysed in this study is in agreement with reports from other species. In fact, reduced PA levels in tobacco and petunia flowers are associated with incomplete or abnormal floral organs [98, 99]. However, additional studies are necessary to confirm these speculations and to explore the relationship between PA biosynthesis and pistil development.

As previously discussed, our data showed that the expression of the *OeFLS*, *OeCHS* and *OeCHI* genes, which are involved in flavonoid biosynthesis, were down-regulated during flower development. A role of flavonoids in ovary abortion has never been demonstrated in olive; however, their actions in reproductive organ development, viability and fertility, as well as in the fate of flower buds have been hypothesized [100, 101, 102]. This pathway is regulated by transcription factors, including proteins of the R2R3-MYB class, some of which act as activators and others as repressor-like proteins [103]. *OeMYBPA1* is reported to act as an activator of this pathway in grape [53], and *OeMYBC2* seems to repress the transcription of genes involved in flavonoid biosynthesis [104, 54, 103]. These genes showed opposite expression trends in olive flowers, and their relative mRNA levels were in accord with the decreased expression of *OeFLS*, *OeCHS* and *OeCHI* genes during flower development. In fact, *OeMYBC2* mRNA levels were significantly higher in perfect open flowers, whereas, those of *OeMYBPA1* were lower. Additional experiments are needed to explain the strong differential expression of these genes in the analysed samples and to examine their involvement in the biological processes of olive flower.

### Transcripts that are putatively involved in pollen-pistil interactions

The analysis of the genes that were differentially expressed between Frantoio (self-compatible) and Leccino (self-incompatible) at anthesis enabled us to identify candidate genes that may be involved in pollen-pistil interactions. Genes related to cell wall modifications, including the glycoside hydrolase families, such as polygalacturonases, β-fructofuranosidase and β-glucosidases, were up-regulated in Leccino flowers. The polygalacturonase activities were associated with a wide range of plant developmental programs, including organ abscission, anther dehiscence, pollen grain maturation and pollen tube growth [105, 106]. It has also been proposed that these enzymes may be involved in the remodelling of the pollen tube cell wall during its growth along the stylar transmitting tissues [107]; nevertheless, our results suggest that they might also have a specific role in the self-incompatible interactions in olive. Recently, Suarez and co-authors [62] reported the spatial and temporal organization of pectins and arabinogalactan proteins (AGPs) in olive pistil during and after pollination. They hypothesized that esterified and de-esterified pectins, arabinose-rich pectins, and arabinogalactan proteins, that are present during pollination, may be involved in pollen-pistil interactions in olive.

Some genes that are involved in programmed cell death (PCD) were down-regulated in Frantoio compared to Leccino. Serrano and co-authors [108, 109] reported that PCD is activated in pollen and stigma papillae during free pollination of olive *in vivo*. However, they found that approximately 80% of pollen undergoes PCD in papillary cells, irrespective of the type of pollen (compatible, incompatible or even sterile), which is apparently in contrast to our results showing that genes related to cell death were expressed at higher levels in the self-incompatible cultivar Leccino after self-pollination.

The up-regulation of genes involved in carbohydrate metabolism might be related to the callose deposition observed in the wall of incompatible pollen grains and tubes, and, in certain cases, in the papillae of stigma following rejection [110]. Moreover, the extracellular secretions produced by the cells of the transmitting tissues are rich in carbohydrates, proteins and lipids, which nourish the growing pollen tubes [111].

The candidate gene approach allowed us to enlarge the set of transcripts may be involved in pollen-pistil interactions. Their expression, as detected by RT-qPCR analyses, is in agreement with their putative involvement in the pollen-pistil recognition process. In fact, orthologs of four lipid transfer proteins (OeLTP1-2-3-4), that are necessary for pollen tube adhesion on the stigma and growth in the stylar transmitting tract [112] and are associated with the accumulation of large amounts of secreted lipids [113], were expressed at significantly higher levels in pistil compared to anthers. In addition, the expression of putative pollen components followed an expected pattern, resulting highly expressed in anthers compared to pistils. Among them, LAT52, secreted by pollen, may function as a bridge to transduce signals into the pollen cytoplasm and plays a role in polarized pollen tube growth [26, 114]. PME1-2 de-esterify the pectin methylester to induce thickening of the apical cell wall and therefore inhibit pollen tube growth [58], whereas, OLE is implicated in pollen hydration and NTP affects pollen tube growth. Finally, βGLU is involved in the callose degradation during the process of tetrad dissolution [57, 26, 115], and caleosin is a component of pollen exine, whose function is still unknown but is thought to act in pollen-stigma signalling. The temporal expression pattern during flower development also support the role of these genes, as their expression increased at full anthesis, just before pollen-pistil interaction occurs, particularly for *OeNTP* and *OePME1* in Frantoio. Considering that the experiments were performed before the pollen meets the stigma, it was not possible to verify eventual changes in the expression of these genes as a result of the reaction of incompatibility.

The RT-qPCR data confirmed the differences between the developmental stages observed by the preliminary computational analyses on entire flowers, however, the differences between the genotypes were not confirmed for *OePM1*, *OePM2* and *OeβGLU*. This might be due the use of entire flowers instead of single flower tissues in the preliminary analyses. Relative differences of mRNA levels among different flower tissues in the two varieties might have produced this discrepancy. These results outline the importance of using separate plant tissues for differential expression studies. Some transcripts highly similar to the female determinants of sporophytic SI system typical of Brassicaceae, such as SRK and SLG, were also identified in our set of flower transcripts. These results support the hypothesis that a Brassica-like sporophytic SI system might occur in olive, as previously proposed [21, 22, 23], even if one transcript with similarity to the pistil S-determinant (*PrsS*) of Papaveraceae was also identified. Putative homologs of important factors involved in the SRK-SCR signalling, including *MLPK* and *EXO70 A1*, were also identified in olive. MLPK is a plasma-membrane-anchored serine-threonine kinase that acts with SRK to transduce SI signals [116, 117]. EXO70 A1 is a subunit of the exocyst complex that has been proposed to function in the polarized delivery of vesicles containing factors that facilitate pollen hydration, germination and growth [118].

Homologs of *PRK*, a receptor kinase required for pollen tube growth [119], interacting with the pollen-specific secreted cysteine-rich protein, LAT52, were also identified in the olive EST libraries, suggesting that this recognition mechanism might also be conserved in olive.

## Conclusions

By sequencing the whole flower transcriptome, performing an ontological annotation of the transcripts in distinct olive genotypes and conducting gene expression studies in different tissues and developmental stages, we were able to identify candidate genes that have crucial functions in a number of biosynthetic pathways involved in flower development and plant reproduction, including the specific biological processes affecting fruit set, such as pre-fertilization, ovary abortion, pollen-pistil interactions and self-incompatibility. Our results represent an important step towards further functional genomics and systems biology research to completely unravel the gene functions and regulatory networks characterizing olive flower and its reproductive barriers. Moreover, our data serve as a valuable source of effective polymorphisms related to these traits that can be used for molecular breeding programs aimed to increase the production.

The elucidation of the molecular and genetic mechanisms of plant reproduction is particularly relevant in fruit tree species, and the knowledge gained in this field may allow us to proceed with tailored initiatives to regulate plant flowering, fruiting and bearing.

## Supporting Information

S1 Fig Functional annotation of the unigenes. A) Percent of unigenes that did or did not match the BLAST hits retrieved from the NCBI databases and their distribution in different plant species. B) Distribution of the unigenes in the following main GO categories: biological processes, cellular component and molecular function.
(TIF)

S2 Fig The MapMan overview maps related to Metabolism, Regulatory Network, Cellular response and Transcription show the differences in the transcript levels between the cvs. Leccino and Dolce Agogia flowers at pre-anthesis (stages 1–6).
(TIF)

S3 Fig MapMan overview. The MapMan overview maps related to Metabolism, Regulatory Network, Cellular response and Transcription show the differences in the transcript levels between the cvs. Frantoio and Leccino flowers at anthesis (stage 7–10).
(TIF)

S4 Fig Phylogenetic tree of flower organ genes. Detailed phylogenetic tree of the floral organ identity determinants based on ABC model. The tree was constructed using the Neighbour-Joining method. The p-distance model and the pairwise deletion option were adopted. A bootstrap analysis was performed by using 1,000 replications. Bootstrap values higher than 75% were indicated.
(TIF)

S1 Table Protein involved in flower development. Key proteins in flower development according to the ABC model that were included in the phylogenetic analysis.
(DOC)

S2 Table Primers used for the RT-qPCR analyses. The primer sequences and amplicon sizes are provided.
(DOC)

S3 Table Functional annotation of flower unigenes. List of all of the olive flower unigenes and their predicted functional annotation. The best hits from the BLASTX search, GO ID, Kegg ID and KO ID are provided.
(XLSX)

S4 Table Genes differentially expressed between pre and post anthesis. List of the differentially expressed transcripts between L1-L6 and L7-L10 and their predicted functional annotation. For each TC, the EST contribution to each genotype or developmental stage is also provided.
(XLSX)

S5 Table Genes differentially expressed in pre anthesis between Leccino and Dolce Agogia. List of the differentially expressed transcripts between L1-L6 and DA1-DA6 and their predicted functional annotation. For each TC, the EST contribution to each genotype or developmental stage is also provided.
(XLSX)

S6 Table Genes differentially expressed in post anthesis between Leccino e Frantoio. List of the differentially expressed transcripts between L7-L10 and F7-F10 and their predicted functional annotation. For each TC, the EST contribution to each genotype or developmental stage is also provided.
(XLSX)

S7 Table Olive transcripts in EST collection. Other olive transcripts identified in the EST collection, putatively involved in ovary abortion and pollen-pistil interactions.
(DOCX)
