# Correction: Transcript Analysis and Regulative Events during Flower Development in Olive (*Olea europaea* L.)

**DOI:** 10.1371/journal.pone.0263101

**Published:** 2022-01-21

**Authors:** Fiammetta Alagna, Marco Cirilli, Giulio Galla, Fabrizio Carbone, Loretta Daddiego, Paolo Facella, Loredana Lopez, Chiara Colao, Roberto Mariotti, Nicolò Cultrera, Martina Rossi, Gianni Barcaccia, Luciana Baldoni, Rosario Muleo, Gaetano Perrotta

Raw data underlying quantitative RT-qPCR results in Figs 5, 6, and 7 of this article [1] are provided with this notice in S1–S3 Files. The raw data underlying *OeAP1*, *OeAP2*, *OeFUL*, *EoSEP2*.*1*, and *OeSEP4* results in Fig 5C are not currently available.

## Supporting information

S1 File Original raw data underlying Fig 5. (XLSX)

S2 File Original raw data underlying Fig 6. (XLSX)

S3 File Original raw data underlying Fig 7. (XLSX)
