# Supplementary figures and images for: Transcript Analysis and Regulative Events during Flower Development in Olive (Olea europaea L.)
Source: PLoS One. 2016 Apr 14;11(4):e0152943. doi: 10.1371/journal.pone.0152943 (PMC4831748; doi:10.1371/journal.pone.0152943)

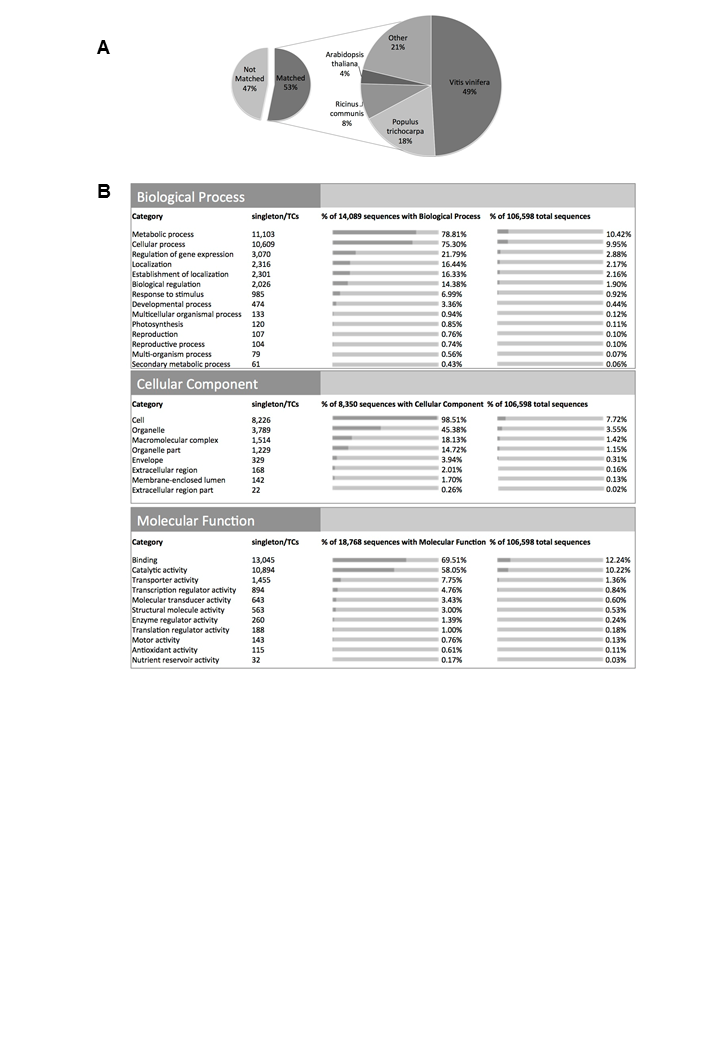

Supplement: S1 Fig — A) Percent of unigenes that did or did not match the BLAST hits retrieved from the NCBI databases and their distribution in different plant species. B) Distribution of the unigenes in the following main GO categories: biological processes, cellular component and molecular function. (TIF) [file pone.0152943.s001.tif]

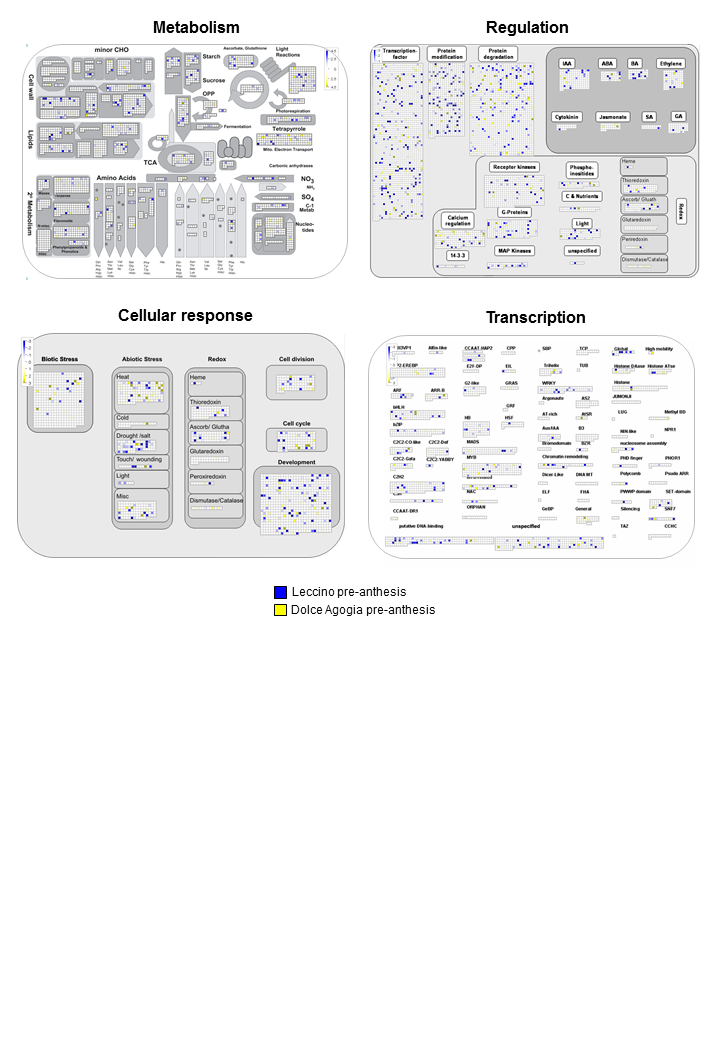

Supplement: S2 Fig — Leccino and Dolce Agogia flowers at pre-anthesis (stages 1–6). (TIF) [file pone.0152943.s002.tif]

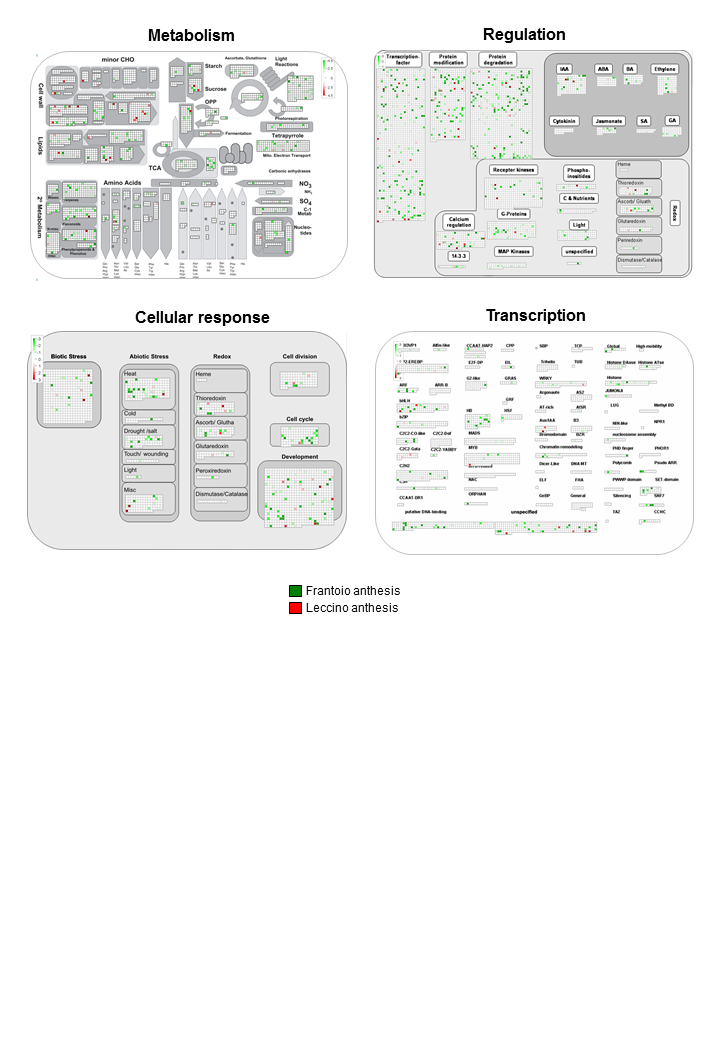

Supplement: S3 Fig — The MapMan overview maps related to Metabolism, Regulatory Network, Cellular response and Transcription show the differences in the transcript levels between the cvs. Frantoio and Leccino flowers at anthesis (stage 7–10). (TIF) [file pone.0152943.s003.tif]

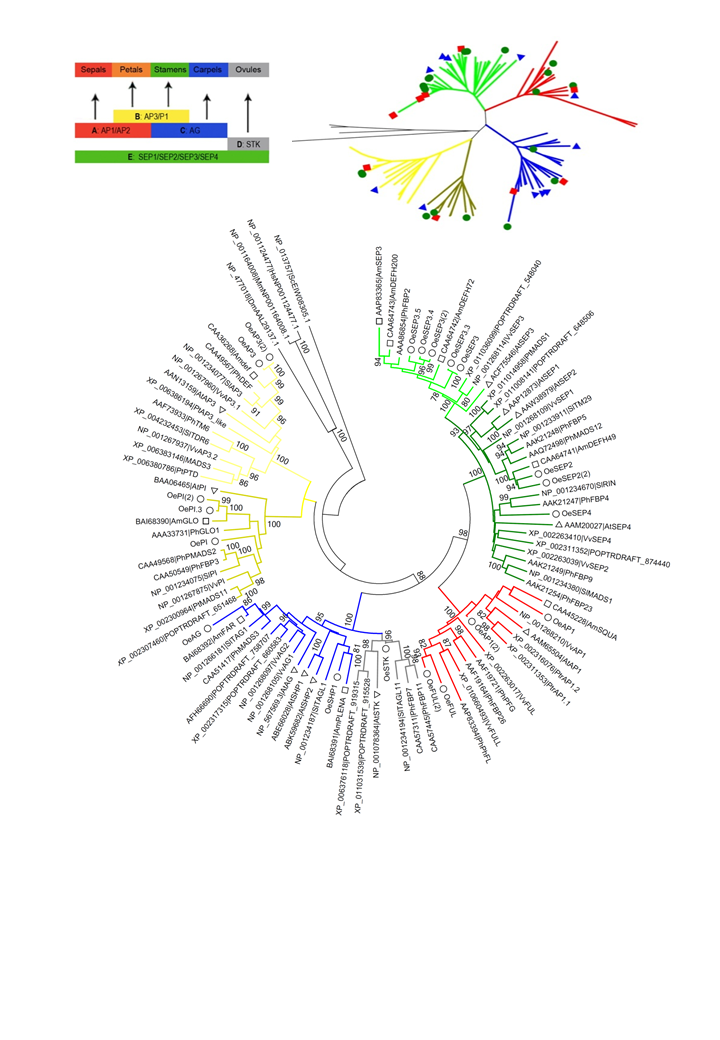

Supplement: S4 Fig — Detailed phylogenetic tree of the floral organ identity determinants based on ABC model. The tree was constructed using the Neighbour-Joining method. The p-distance model and the pairwise deletion option were adopted. A bootstrap analysis was performed by using 1,000 replications. Bootstrap values higher than 75% were indicated. (TIF) [file pone.0152943.s004.tif]
