# Supplementary material for: Transcript Analysis and Regulative Events during Flower Development in Olive (Olea europaea L.)
Source: PLoS One. 2016 Apr 14;11(4):e0152943. doi: 10.1371/journal.pone.0152943 (PMC4831748; doi:10.1371/journal.pone.0152943)
Supplement: S1 Table — Key proteins in flower development according to the ABC model that were included in the phylogenetic analysis. (DOC) [file pone.0152943.s005.doc]

**Supplementary Table S1. Key proteins of flower development according to the ABC model included in phylogenetic analysis.**

| **Protein** | **Species** | **Accession number** |
| --- | --- | --- |
| Amdef | *Antirrhinum majus* | CAA36268.1 |
| AmDEFH200 | *Antirrhinum majus* | CAA64743.1 |
| AmDEFH49 | *Antirrhinum majus* | CAA64741.1 |
| AmDEFH72 | *Antirrhinum majus* | CAA64742.1 |
| AmFAR | *Antirrhinum majus* | BAI68392.1 |
| AmGLO | *Antirrhinum majus* | BAI68390.1 |
| AmPLENA | *Antirrhinum majus* | BAI68391.1 |
| AmSEP3 | *Antirrhinum majus* | AAP83365.1 |
| AmSQUA | *Antirrhinum majus* | CAA45228.1 |
| AtAG | *Arabidopsis thaliana* | NP_567569.3 |
| AtAP1 | *Arabidopsis thaliana* | AAM65504.1 |
| AtAP3 | *Arabidopsis thaliana* | AAN13159.1 |
| AtPI | *Arabidopsis thaliana* | BAA06465.1 |
| AtSEP1 | *Arabidopsis thaliana* | AAP12873.1 |
| AtSEP2 | *Arabidopsis thaliana* | AAW38979.1 |
| AtSEP3 | *Arabidopsis thaliana* | ACF75546.1 |
| AtSEP4 | *Arabidopsis thaliana* | AAM20027.1 |
| AtSHP1 | *Arabidopsis thaliana* | ABE66028.1 |
| AtSHP2 | *Arabidopsis thaliana* | ABK59682.1 |
| AtSTK | *Arabidopsis thaliana* | NP_001078364.1 |
| PhDEF | *Petunia x hybrida* | CAA49567.1 |
| PhFBP11 | *Petunia x hybrida* | CAA57445.1 |
| PhFBP2 | *Petunia x hybrida* | AAA86854.1 |
| PhFBP23 | *Petunia x hybrida* | AAK21254.1 |
| PhFBP26 | *Petunia x hybrida* | AAF19164.1 |
| PhFBP3 | *Petunia x hybrida* | CAA50549.1 |
| PhFBP4 | *Petunia x hybrida* | AAK21247.1 |
| PhFBP5 | *Petunia x hybrida* | AAK21248.1 |
| PhFBP7 | *Petunia x hybrida* | CAA57311.1 |
| PhFBP9 | *Petunia x hybrida* | AAK21249.1 |
| PhGLO1 | *Petunia x hybrida* | AAA33731.1 |
| PhMADS12 | *Petunia x hybrida* | AAQ72498.1 |
| PhMADS3 | *Petunia x hybrida* | CAA51417.1 |
| PhPFG | *Petunia x hybrida* | AAF19721.1 |
| PhPhFL | *Petunia x hybrida* | AAP83394.1 |
| PhPMADS2 | *Petunia x hybrida* | CAA49568.1 |
| PhTM6 | *Petunia x hybrida* | AAF73933.1 |
| POPTRDRAFT_548040 | *Populus euphratica* | XP_011036099.1 |
| POPTRDRAFT_648506 | *Populus euphratica* | XP_011008141.1 |
| POPTRDRAFT_651468 | *Populus trichocarpa* | XP_002307460.2 |
| POPTRDRAFT_660583 | *Populus trichocarpa* | XP_002317315.1 |
| POPTRDRAFT_758707 | *Populus simonii x Populus nigra* | AFH66690.1 |
| POPTRDRAFT_874440 | *Populus trichocarpa* | XP_002311352.1 |
| POPTRDRAFT_915528 | *Populus euphratica* | XP_011031539.1 |
| POPTRDRAFT_919315 | *Populus trichocarpa* | XP_006376118.1 |
| PtAP3_like | *Populus trichocarpa* | XP_006386194.1 |
| PtMADS1 | *Populus euphratica* | XP_011014958.1 |
| PtMADS11 | *Populus trichocarpa* | XP_002300964.1 |
| PtPTD | *Populus trichocarpa* | XP_006380786.1 |
| PtrAP1.1 | *Populus trichocarpa* | XP_002311353.2 |
| PtrAP1.2 | *Populus trichocarpa* | XP_002316076.1 |
| MADS3 | *Populus trichocarpa* | XP_006383146 |
| ScEIW08305.1 | *Saccharomyces cerevisiae* | NP_013757 |
| DmAAL29137.1 | *Drosophila melanogaster* | NP_477018 |
| MmNP001164008.1 | *Mus musculus* | NP_001164008 |
| HsNP001124477.1 | *Homo sapiens* | NP_001124477 |
| SlAP3 | *Solanum lycopersicum* | NP_001234077 |
| SlDEF | *Solanum lycopersicum* | NP_001234077 |
| SlMADS1 | *Solanum lycopersicum* | NP_001234380 |
| SlPI | *Solanum lycopersicum* | NP_001234075 |
| SlRIN | *Solanum lycopersicum* | NP_001234670 |
| SlTAG1 | *Solanum lycopersicum* | NP_001266181 |
| SlTAGL1 | *Solanum lycopersicum* | NP_001234187 |
| SlTAGL11 | *Solanum lycopersicum* | NP_001234194 |
| SlTAP3 | *Solanum lycopersicum* | NP_001234077 |
| SlTDR6 | *Solanum lycopersicum* | XP_004232453 |
| SlTM29 | *Solanum lycopersicum* | NP_001233911 |
| VvAG1 | *Vitis vinifera* | NP_001268105 |
| VvAG2 | *Vitis vinifera* | NP_001268097 |
| VvAP1 | *Vitis vinifera* | NP_001268210 |
| VvAP3.1 | *Vitis vinifera* | NP_001267960 |
| VvAP3.2 | *Vitis vinifera* | NP_001267937 |
| VvFUL | *Vitis vinifera* | XP_002263017 |
| VvFULL | *Vitis vinifera* | XP_010660493 |
| VvPI | *Vitis vinifera* | NP_001267875 |
| VvSEP1 | *Vitis vinifera* | NP_001268109 |
| VvSEP2 | *Vitis vinifera* | XP_002263039 |
| VvSEP3 | *Vitis vinifera* | NP_001268114 |
| VvSEP4 | *Vitis vinifera* | XP_002263410 |
| OeAG | *Olea europaea* | Present research |
| OeAP1.1 | *Olea europaea* | Present research |
| OeAP1.2 | *Olea europaea* | Present research |
| OeAP3.1 | *Olea europaea* | Present research |
| OeAP3.2 | *Olea europaea* | Present research |
| OeFUL.1 | *Olea europaea* | Present research |
| OeFUL.2 | *Olea europaea* | Present research |
| OePI.1 | *Olea europaea* | Present research |
| OePI.2 | *Olea europaea* | Present research |
| OePI.3 | *Olea europaea* | Present research |
| OeSEP2.1 | *Olea europaea* | Present research |
| OeSEP2.2 | *Olea europaea* | Present research |
| OeSEP3.1 | *Olea europaea* | Present research |
| OeSEP3.2 | *Olea europaea* | Present research |
| OeSEP3.3 | *Olea europaea* | Present research |
| OeSEP3.4 | *Olea europaea* | Present research |
| OeSEP3.5 | *Olea europaea* | Present research |
| OeSEP4.1 | *Olea europaea* | Present research |
| OeSHP1 | *Olea europaea* | Present research |
| OeSTK | *Olea europaea* | Present research |
