# Supplementary material for: Transcript Analysis and Regulative Events during Flower Development in Olive (Olea europaea L.)
Source: PLoS One. 2016 Apr 14;11(4):e0152943. doi: 10.1371/journal.pone.0152943 (PMC4831748; doi:10.1371/journal.pone.0152943)
Supplement: S2 Table — The primer sequences and amplicon sizes are provided. (DOC) [file pone.0152943.s006.doc]

**Supplementary Table S2. Primers used for RT-qPCR analyses of transcripts.**

| **Transcript** | **Primer forward** | **Primer reverse** | **Amplicon size (bp)** |
| --- | --- | --- | --- |
| *OeAP1* | CAAAGAGATGGAGAAGGAAATG | ATTACCGCCATGATTAGGATTA | 70 |
| *OeAP2* | AATGCCGTTTGAATTTGATTTG | ATTATATCCATCCCTTCTGTCA | 97 |
| *OeFUL* | TGATTGTATGTTCTGGGCTTAAC | CGTATATCTGCTTGTGAAAGGT | 73 |
| *OeAP3* | TCATCACCCTAATCTTCACAGT | TTCTTCAAACACAACGGTCTTT | 146 |
| *OePI* | ATCTCCGTGAAAGAATTTAGGAAA | CCATCTATAAAAAGAGGTAGCAGAA | 99 |
| *OeAG* | CGACTTACATCATAACAATCAG | TCTTGGGTAGGGTAATCATC | 163 |
| *OeAG* | CTTCGAGGAAAAATGGAAGAGG | CAGCGTCACACAACACAGACA | 135 |
| *OeSHP1* | GACAGCTCCCTTATCAAAGTTA | CAATAGAAGAACGAAGCCGA | 72 |
| *OeSTK* | ACTGATTGTTTTCTCTAGCCGT | TTGTTGGTAGAACTGAGCGTTAAT | 145 |
| *OeSEP2* | GCAGACTTTCACCAGCAATTAT | ATACACTCGATTTCGTTTAAGTCA | 117 |
| *OeSEP2.2* | TTACGATTCAAGAATCCTCATGCT | CAATAGATATAACAGAAAGATTCACCC | 85 |
| *OeSEP3* | GTTGCCTTGAACATGAGAA | GTGCTTGAGAGAAATCCATAC | 115 |
| *OeSEP4* | GATTTCAGCCTCCTCAGATTG | CTGTTATCCGATGCCACATAAT | 94 |
| *OeEF1a* | CTGACTGCGCCGTCCTTATC | TGACACCAAGGGTGAAGGC | 111 |
| *OeLAT52* | CGATTGTAGTGTGGCAATGG | AGTGGGTTTGCGTAACGAAC | 96 |
| *OeNTP* | GTGTGGTATCGTCCGTGGTT | GCCATGCTTTGTTCTTCCAT | 200 |
| *OLEOSIN* | CACTCCCTTGCTGGTCATTT | GCAGTTGGTCGTTTTCCAGT | 159 |
| *OeβGLU* | ACTTTCAACATGCCGGAAGAA | TTTCAGAACCACTTCGGAAACT | 182 |
| *OePME1* | GCTTTCCGAACAGCTACTGG | CGTGGAGGCAGTTCTATTGG | 138 |
| *OePME2* | ACGCGTTTAGCATTTCCATC | CGCCAGACAGTGCTGAAATA | 199 |
| *OeLTP1* | TTTCATCAGTGGAGCAGAGAGTT | CCAGGGTTTTCCCTTCACTC | 102 |
| *OeLTP2* | TTTCATCGCGAATGGAGTTT | CGGATTACAGACAAAATGCAGA | 132 |
| *OeLTP3* | GAGGTTTCTGCTGTGGCCTA | AAAACGCCAGGTTTTCTCTACA | 105 |
| *OeLTP4* | TGGCTGCTCTACCTGAAGGA | CAACAGAGAGGATAACGCGAAC | 101 |
| *OeSUSy* | GCCTGGACTCTACCGAGTTGTT | CACGCATAGGTGTTCCTTGTTC | 187 |
| *OeInv-V* | CCAGTCAGCGAAGTGGAAGAAT | TGTAACCAGCATCAGCATCAGC | 184 |
| *OeInv-CW* | AGACAAGGCAGAGACATTCGAC | ATGCATCAGAGCACATGAGAAC | 206 |
| *OeGBSSI* | TGTGCCAAAGTCGACCCTGCCG | TGGTTCACTGCTGGCAGCCCC | 125 |
| *OeBAM1* | TGCCACGATATGATGACTACGC | TCAGGTTGGAACAAATCCGGGTTC | 134 |
| *OeARG* | GATGTGGACTGTCTGGATCCGGCA | CCATCAACAGTATCGCGCTGCGG | 161 |
| *OeNLP1* | TTAGCTTCTTTGAGGAGGCAAAC | TGGACCATCTGGTATATGGGACT | 110 |
| *OeSPMS* | TATGGATGGGAGGGAGACTGA | GCCACATTGGGTTGTTGAAATA | 197 |
| *OeSPDS* | GGTTCTGTGAACTACGCTTGGA | CAGCATCTATTGGGTTGATTGG | 124 |
| *OeMYBPA1* | AACGAAGAAGGAAAGGAACGGTAC | CAGACGATGCAGCACTCAACAAAC | 131 |
| *OeMYBC2* | GGAAACTTTTGAGCAGAGGTATTG | CCGGGATAATTTCTTCTTTGTTGG | 168 |
| *OeActin7a* | GGTTGGGATGGGACAGAAGGATGC | TGAGAGGTGCCTCAGTGAGAAGCA | 199 |
