# Supplementary material for: Transcript Analysis and Regulative Events during Flower Development in Olive (Olea europaea L.)
Source: PLoS One. 2016 Apr 14;11(4):e0152943. doi: 10.1371/journal.pone.0152943 (PMC4831748; doi:10.1371/journal.pone.0152943)
Supplement: S7 Table — Other olive transcripts identified in the EST collection, putatively involved in ovary abortion and pollen-pistil interactions. (DOCX) [file pone.0152943.s011.docx]

**S7 Table. Other olive transcripts identified in the EST collection, putatively involved in ovary abortion and pollen-pistil interactions.**

| **Gene^a^** | **EST ID^b^** | **BLAST best match** | **Length (bp)** | **E-value^c^** | **Function** | **References** | |
| --- | --- | --- | --- | --- | --- | --- | --- |
| **Olive transcripts putatively involved in ovary abortion** | | | | | | |  |
| *S-adenosylmethionine decarboxylase* (*SAMDC*) | unigene00654  unigene00546  unigene00546 | [*Olea europaea*] GU190157.1 | 1,751  1,019  734 | 0.0  1e^-68^  1e^-105^ | Polyamine biosynthesis | Gomez-Jimenez et al. 2010 | |
| *Polygalacturonase-like Glycosyl idrolase family 28* | unigene01310  unigene01077  unigene01547  F7KHMQ102EZN4J | [*Solanum lycopersicum*] XP_004243884.1 | 1,476  1,705  1,448  320 | 9e^-169^  2e^-162^  6e^-169^  5e^-13^ | Cell wall metabolism | Rejon et al. 2013 | |
| *Metacaspase type I* | F7KHMQ104IAVWT  unigene01138 | [*Nicotiana tabacum*] ACG59943.1 | 248  1,236 | 2e^-50^  2e^-139^ | Programmed cell death | Sanmartin et al. 2005 | |
| *Cysteine protease* | unigene01194  F7KHMQ102D8GWX  unigene09327 | [*Ricinus communis*] EEF37274.1 | 387  1,592  564 | 3e^-107^  2e^-69^  2e^-11^ | Programmed cell death | Shahri and Tahir 2014 | |
| *PLENA/AGAMOUS* (*AG*) | unigene03887 | [*Antirrhinum majus*] AAB25101.1 | 963 | 2e^-118^ | MADS-box flower development | Davies et al. 1999; Tadiello et al. 2009 | |
| *Chalcone synthase* (*CHS*) | unigene01976  unigene01823  unigene07780  F7KHMQ101B3PAZ  F7KHMQ104IBHTU | [*Ricinus communis*] EEF33135.1 | 1,460  1,472  321  431  403 | 0.0  0.0  2e^-30^  6e^-34^  6e-^25^ | Flavonoid pathway | Napoli et al. 1999 | |
| *Flavonol synthase* (*FLS*) | unigene02434  unigene02473  unigene02434  unigene02203  F7KHMQ101AVVC8 | [*Petunia* x *hybrida*] CAA80264.1 | 1,198  1,226  512  991  88 | 0.0  4e^-180^  7e^-54^  2e^-150^  2e^-24^ | Flavonoid pathway | Holton et al. 1993; Van Eldik et al. 1997 | |
| **Olive transcripts putatively involved in pollen-pistil interactions** | | | | | | |  |
| S receptor kinase (SRK) | unigene02160 | [*Brassica oleracea*] M76647.1 | 1,241 | 1e^-130^ | Female determinant of self-incompatibility | Stein et al. 1991; Takayama and Isogai 2005 | |
|  | unigene02300 |  | 1,215 | 6e^-087^ |  |  |  |
|  | unigene01237 |  | 1,488 | 1e^-071^ |  |  |  |
|  | unigene02831 |  | 1,124 | 1e^-068^ |  |  |  |
|  | unigene00597 |  | 1,740 | 7e^-064^ |  |  |  |
|  | F7KHMQ104IWT7W |  | 522 | 2e^-063^ |  |  |  |
|  | unigene01626 |  | 1,369 | 1e^-062^ |  |  |  |
|  | unigene07245 |  | 693 | 9e^-061^ |  |  |  |
|  | unigene07615 |  | 671 | 2e^-058^ |  |  |  |
|  | F7KHMQ102DBVEU |  | 491 | 1e^-057^ |  |  |  |
|  | unigene08988 |  | 579 | 2e^-057^ |  |  |  |
|  | unigene03520 |  | 1,028 | 3e^-58^ |  |  |  |
|  | F7KHMQ102EJ7HP |  | 498 | 8e^-054^ |  |  |  |
|  | F7KHMQ101AVTHL |  | 470 | 2e^-049^ |  |  |  |
|  | F7KHMQ103FU71M |  | 464 | 8e^-048^ |  |  |  |
| *S-locus glycoprotein* (*SLG*) | F7KHMQ102DKTLC | [*Brassica oleracea*] CAA38995 | 516 | 9e^-035^ | Enhancer the activity of the receptor of SCR/SP11 | Dwyer et al. 1991; Takayama and Isogai 2005 | |
|  | F7KHMQ103G1GJL |  | 501 | 1e^-030^ |  |  |  |
|  | F7KHMQ102D1V7H |  | 521 | 7e^-030^ |  |  |  |
|  | F7KHMQ104I0SEV |  | 515 | 3e^-029^ |  |  |  |
|  | F7KHMQ102EHUI8 |  | 512 | 5e^-029^ |  |  |  |
|  | F7KHMQ102ES0LD |  | 505 | 8e^-028^ |  |  |  |
|  | F7KHMQ104IIKK5 |  | 508 | 2e^-026^ |  |  |  |
|  | F7KHMQ102EBES4 |  | 520 | 3e^-026^ |  |  |  |
|  | F7KHMQ104JDUN1 |  | 514 | 2e^-024^ |  |  |  |
|  | F7KHMQ102EZ42A |  | 477 | 6e^-023^ |  |  |  |
|  | F7KHMQ101CGTRN |  | 501 | 7e^-023^ |  |  |  |
|  | F7KHMQ104JP7VJ |  | 490 | 1e^-021^ |  |  |  |
|  | unigene07199 |  | 687 | 3e^-020^ |  |  |  |
|  | F7KHMQ104IDFRZ |  | 513 | 4e^-020^ |  |  |  |
|  | F7KHMQ102DF3EI |  | 522 | 2e^-019^ |  |  |  |
| *Pistil S-determinant* (*PrsS*) | F7KHMQ102DJPG4 | [*Papaver rhoeas*] X74333 | 458 | 1e^-21^ | Female determinant of self-incompatibility | Foote et al. 1994; Wheeler et al. 2009 | |
| *M-locus protein kinase isoform2* (*MLPK*) | unigene02886 | [*Brassica rapa*] ABP01775.1 | 1,116 | 1e^-117^ | Involved in self-incompatibility signalling | Murase et al. 2004; Kakita et al. 2007 | |
|  | unigene01180 |  | 1,512 | 1e^-114^ |  |  |  |
|  | unigene00678 |  | 1,724 | 1e^-104^ |  |  |  |
|  | F7KHMQ104JKHEA |  | 411 | 6e^-80^ |  |  |  |
|  | unigene09185 |  | 567 | 2e^-94^ |  |  |  |
| *Exocyst subunit* (*EXO70 A1*) | unigene00438 | [*Brassica napus*] ACV92697.1 | 1,867 | 0.0 | Self-incompatibility response in Brassicaceae | Samuel et al. 2009 | |
|  | F7KHMQ103G1BAY |  | 479 | 4e^-86^ |  |  |  |
|  | F7KHMQ102C9Q3S |  | 468 | 2e^-86^ |  |  |  |
|  | F7KHMQ103HFC8Q |  | 263 | 3e^-33^ |  |  |  |
| *Receptor-like protein kinase* (*PRK1*) | F7KHMQ101A1GJ3 | [*Lycopersicum esculentum*] AAC12254.1 | 492 | 5e^-042^ | Receptor kinase required for pollen tube growth | Muschietti et al. 1998; Zhang et al, 2008; Kumar and McClure 2010 | |
|  | F7KHMQ103GBX6L |  | 409 | 1e^-041^ |  |  |  |
|  | F7KHMQ103GZ5WW |  | 529 | 1e^-038^ |  |  |  |
|  | F7KHMQ103GOGQH |  | 529 | 2e^-038^ |  |  |  |
|  | F7KHMQ103F1SYQ |  | 449 | 6e^-037^ |  |  |  |
| *Plantacyanin* | unigene03462 | [*Arabidopsis thaliana*] AAC32449.1 | 1,042 | 5e^-014^ | Involved in pollen tube growth | Dong et al. 2005; Higashiyama 2010 | |
|  | unigene06877 |  | 725 | 3e^-013^ |  |  |  |
|  | F7KHMQ101BKB5I |  | 244 | 5e^-013^ |  |  |  |
|  | F7KHMQ101B863T |  | 303 | 2e^-012^ |  |  |  |
|  | F7KHMQ101BKTLT |  | 367 | 2e^-011^ |  |  |  |
|  | unigene07294 |  | 407 | 6e^-011^ |  |  |  |
| *Caleosin* | unigene04323 | [*Arabidopsis thaliana*] NP_173738 | 946 | 2e^-042^ | Putatively involved in pollen germination | Murphy 2006; Zienkiewicz et al. 2010 | |
|  | F7KHMQ102EJ1Y6 |  | 478 | 3e^-029^ |  |  |  |

^a^Name, species and accession number of the genes that were used as reference sequences for the tBLASTx search.

^b^Best hits of the olive transcripts, as identified using a bi-directional BLAST search.

^c^E-value of the best hits identified by the BLASTX search.
